# Influence of Functionalization on the Textural Properties and Photocatalytic Performance of ZnO-Modified Metakaolin Based-Geopolymer

**DOI:** 10.3390/polym18091110

**Published:** 2026-04-30

**Authors:** Adriana-Gabriela Schiopu, Mihai Oproescu, Ștefan Mira, Sorin Georgian Moga, Ecaterina Magdalena Modan, Paul Mereuță, Miruna-Adriana Ioța, Alexandru Berevoianu

**Affiliations:** 1 Doctoral School Materials Science and Engineering, National University of Science and Technology Politehnica Bucharest, Splaiul Independentei No. 313, Sector 6, 060042 Bucharest, Romania; gabriela.schiopu@upb.ro (A.-G.S.); stefan.mira@stud.sed.upb.ro (Ș.M.); iota.miruna@imnr.ro (M.-A.I.); 2 Faculty of Mechanics and Technology, Pitesti University Centre, National University of Science and Technology Politehnica Bucharest, 110040 Pitesti, Romania; 3 Faculty of Electronics, Communication and Computers, Pitesti University Centre, National University of Science and Technology Politehnica Bucharest, 110040 Pitesti, Romania; 4 Regional Center of Research & Development for Materials, Processes and Innovative Products Dedicated to the Automotive Industry (CRCD-AUTO), Pitesti University Centre, National University of Science and Technology Politehnica Bucharest, 060042 Bucharest, Romaniaecaterina.modan@upb.ro (E.M.M.); 5 Horia Hulubei National Institute for R&D in Physics and Nuclear Engineering (IFIN-HH), 077125 Magurele, Romania; paul.mereuta@nipne.ro (P.M.);; 6 National R&D Institute for Non-Ferrous and Rare Metals IMNR, 077145 Pantelimon, Romania; 7 Faculty of Physics, University of Bucharest, 077125 Magurele, Romania

**Keywords:** ZnO-modified metakaolin geopolymers, photocatalytic activity, surface functionalization

## Abstract

Metakaolin-based geopolymers modified with ZnO and surface-functionalized ZnO were developed and investigated in terms of structure, morphology, textural properties, and photocatalytic performance. ZnO, ZnO@AS(zinc oxide functionalized with steric acid), and ZnO@PEG(zinc oxide functionalized with polyethylene glycol) were incorporated into the geopolymer matrix and characterized by XRD(X-ray diffraction), ATR–FTIR(Fourier transform infrared spectroscopy in attenuated total reflectance, SEM–EDS (scanning electron microscopy coupled with spectroscopy with energy-dispersive X-ray spectroscopy), and BET(Brunauer–Emmett–Teller) analysis. The results showed that ZnO incorporation did not significantly modify the amorphous aluminosilicate network but affected the morphology and porosity depending on the functionalization method. ZnO@AS induced matrix densification and reduced accessible porosity, while ZnO@PEG improved particle dispersion and preserved the porous structure. Among the ZnO-modified metakaolin-based geopolymers, GP/ZnO@PEG(geopolymer with ZnO@PEG particles) exhibited the highest photocatalytic performance, characterized by a BET surface area of 17.22 m^2^/g, an apparent kinetic constant of 0.01668 min^−1^, and a half-life of approximately 41 min, achieving approximately 90% methylene blue removal after 120 min of UV-A irradiation. The study demonstrates that ZnO surface functionalization controls the interfacial interaction with the geopolymer matrix and plays a key role in the performance of geopolymer-based photocatalytic materials.

## 1. Introduction

Geopolymers are inorganic aluminosilicate materials synthesized via the alkaline activation of Si- and Al-rich precursors, such as metakaolin or industrial by-products. The geopolymerization process involves dissolution in an alkaline medium followed by polycondensation into a three-dimensional –Si–O–Al–O– network, resulting in high mechanical strength, chemical stability, and thermal resistance [1,2,3].

Owing to their reduced environmental footprint and the valorization of secondary resources, geopolymers are regarded as sustainable alternatives to conventional cement-based materials. Their tunable porosity, adsorption capacity, and compatibility with inorganic phases or nanoparticles enable the development of advanced functional composites [4].

The incorporation of semiconductor metal oxides into geopolymer matrices has attracted significant attention over the past decade, as it enables the development of advanced materials with photocatalytic, antibacterial, and optoelectronic functionalities [5,6,7].

Among these, zinc oxide (ZnO) is one of the most versatile oxides due to its wide band gap (~3.3 eV), high charge carrier mobility, and ability to generate reactive oxygen species under UV irradiation [8,9,10]. ZnO can enhance photocatalytic performance, improve UV stability, and promote the degradation of organic pollutants, such as synthetic dyes [11,12,13,14,15,16,17,18,19,20].

The incorporation of ZnO nanoparticles into geopolymer matrices can be achieved either by direct dispersion or post-synthesis impregnation; however, their effectiveness strongly depends on particle distribution, agglomeration degree, and interaction with the inorganic network [21,22,23,24].

Studies investigating the relationship between internal structure, functionalization-induced modifications, and performance in organic pollutant degradation processes are essential for optimizing these composites and expanding their applicability in water remediation and environmental protection [22]. Previous studies demonstrated that geopolymers can serve as efficient supports for semiconductor photocatalysts such as TiO_2_, ZnO, or ZnTiO_3_, enabling the combined mechanisms of adsorption and photocatalytic degradation of organic dyes [23,24]. However, most investigations focused on the effect of ZnO loading or geopolymer composition on photocatalytic performance, while the role of surface functionalization and interfacial compatibility between ZnO particles and the geopolymer matrix remains insufficiently explored.

Surface functionalization of metal oxide nanoparticles plays a critical role in controlling their dispersion, interfacial interactions, and overall performance in composite systems. In the case of highly alkaline inorganic matrices such as geopolymers, this aspect becomes particularly important due to the presence of aqueous phases, high ionic strength, and complex aluminosilicate networks, which can promote particle agglomeration and limit the accessibility of active sites. The surface polarity of nanoparticles, governed by hydrophobic or hydrophilic functionalization, significantly influences their interaction with both the liquid phase and the solid matrix. Hydrophobic modification reduces surface energy and limits interaction with water, potentially leading to decreased dispersion but enhanced resistance to moisture-induced effects. In contrast, hydrophilic functionalization, for example, using polymers, promotes steric stabilization, improves compatibility with the aqueous phase, and facilitates a more uniform distribution within the matrix. Although surface modification of ZnO has been widely investigated in polymeric and cementitious systems, the comparative effect of hydrophobic versus hydrophilic functionalization in geopolymer matrices remains insufficiently explored. Understanding how surface chemistry influences particle dispersion, pore accessibility, and photocatalytic behavior is therefore essential for optimizing the performance of ZnO–geopolymer composites.

The present study aims to investigate how surface functionalization of ZnO particles influences their dispersion, interfacial interaction, and photocatalytic behavior when incorporated into a metakaolin-based geopolymer matrix. Two different functionalization strategies—hydrophobic stearic acid (ZnO@AS) and hydrophilic polyethylene glycol (ZnO@PEG)—were employed to modulate the particle–matrix interface.

A comprehensive structural and functional correlation was established by combining XRD, SEM-EDS, BET textural analysis, and photocatalytic degradation tests of methylene blue under UV-A irradiation.

The novelty of this work consists of elucidating the role of surface functionalization in controlling the dispersion, porosity, and photocatalytic performance of ZnO-modified geopolymers. By comparing hydrophobic (stearic acid) and hydrophilic (PEG) functionalization routes, the study demonstrates that interfacial compatibility and accessible active surface area are the key factors governing photocatalytic efficiency.

## 2. Materials and Methods

### 2.1. Materials

Surface functionalization of ZnO particles represents an effective strategy to improve particle dispersion, reduce agglomeration, and enhance compatibility with inorganic matrices. Organic modifiers can alter the surface energy, hydrophilicity, and interaction mechanisms between ZnO particles and the geopolymeric matrix, potentially influencing the microstructure and functional properties. The functionalization of the ZnO particles was achieved using two different organic agents, namely stearic acid (AS) and polyethylene glycol (PEG), to modify the surface properties of the nanoparticles and improve their dispersion in the geopolymer matrix. The choice of stearic acid and PEG was based on their complementary surface modification mechanisms. Stearic acid provides hydrophobic functionalization through carboxylate bonding to ZnO, reducing surface energy and limiting interaction with aqueous media, while PEG offers hydrophilic steric stabilization, improving dispersion and accessibility of active sites. This dual approach was intentionally selected to compare the influence of surface polarity on ZnO behavior in geopolymer matrices, as supported by previous studies on fatty acid and polymer functionalization of metal oxide nanoparticles. Stearic acid is a low-molecular-weight fatty acid that interacts with the ZnO surface through carboxylate–Zn bonding, forming a relatively compact hydrophobic layer. Due to its small molecular size and the need to ensure sufficient surface interaction, a higher mass ratio was employed to achieve a noticeable modification of surface energy and interparticle interactions. In contrast, PEG 4000 is a high-molecular-weight polymer that acts primarily through steric stabilization and hydrogen bonding, forming a flexible hydrophilic layer on the ZnO surface. Because of its polymeric nature, even a relatively small mass is sufficient to influence dispersion behavior and interfacial compatibility. Increasing the PEG content excessively may lead to partial shielding of active sites or formation of a thick polymer layer, which could negatively affect photocatalytic performance.

In the case of stearic acid functionalization, 0.25 g of stearic acid (Sigma-Aldrich, St. Louis, MO, USA) was dissolved in 20 mL of ethanol, the solution being maintained at 60 °C under magnetic agitation until a homogeneous liquid phase was obtained. Subsequently, 0.5 g of ZnO powder (Sigma-Aldrich, St. Louis, MO, USA) was added to the resulting solution and the suspension was kept under agitation for 20 min to allow the adsorption of stearic acid molecules on the surface of the zinc oxide particles. After the homogenization step, the solvent was removed by thermal evaporation at 80 °C for 3 h, obtaining the ZnO powder functionalized with stearic acid, denoted ZnO@AS.

The functionalization with polyethylene glycol was achieved by a similar method, using a hydroalcoholic medium. Thus, 0.05 g of PEG 4000 (Sigma-Aldrich, St. Louis, MO, USA) was dissolved in 35 mL of 70% ethanol solution, until a homogeneous solution was obtained. 0.5 g of ZnO powder was added to this solution, and the resulting suspension was subjected to sonication for 30 min to ensure efficient particle dispersion and uniform interaction between the polymer chains and the zinc oxide surface. Subsequently, the solvent was removed by evaporation in an oven at 50 °C for 3 h, followed by additional heat treatment at 100 °C for 2 h to completely remove the residual solvent and stabilize the polymer layer on the particle surface. The resulting material was denoted ZnO@PEG.

Therefore, the selected quantities were chosen as practical working ratios to generate two distinct and contrasting surface chemistries—hydrophobic (ZnO@AS) and hydrophilic (ZnO@PEG)—allowing a comparative investigation of the effect of surface polarity on dispersion, porosity, and photocatalytic behavior in geopolymer matrices. The objective was not to achieve identical surface coverage, but to obtain clearly differentiated interfacial conditions.

**Figure 1:**
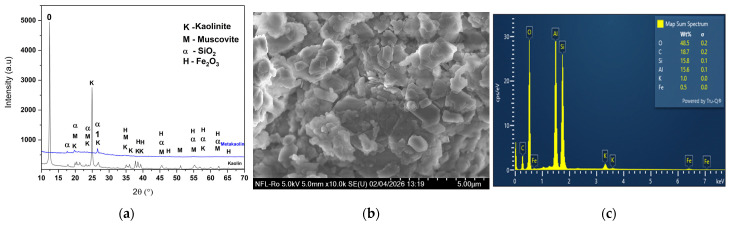
XRD of (**a**) kaolin and metakaolin, (**b**) SEM image and (**c**) EDS analysis of metakaolin.

For the synthesis of GP (geopolymers) and ZnO-modified GP, metakaolin is obtained by calcination of kaolin (Naturall Hom, Salonta, Romania) at 750 °C for 2 h in a Mikrotest MKF-05 muffle furnace (Ankara, Türkiye) and was used as the aluminosilicate precursor. The XRD characterization of kaolin and metakaolin is presented in Figure 1a. The transformation of kaolinite (K) into an amorphous metakaolin (M) phase is evidenced by the disappearance of characteristic kaolinite peaks and the appearance of a broad amorphous halo. Minor crystalline phases quartz (α-SiO_2_) and hematite (H-Fe_2_O_3_) are also identified (details in Supplementary File S1).

The SEM-EDS analysis of metakaolin is presented in Figure 1b,c. The particle size analysis, using ImageJ software version 1.54d, revealed an average value of 1.86 μm, with a relatively high standard deviation of 1.11 μm. This indicates a broad particle size distribution, suggesting a significant degree of heterogeneity and possible agglomeration phenomena within the metakaolin powder. The initial Si/Al molar ratio was calculated to be approximately 0.97, indicating a nearly equimolar distribution of Si and Al species in the metakaolin precursor.

The alkaline activator consisted of 3.6 g of 8 M KOH (Sigma-Aldrich, St. Louis, MO, USA) and 3.6 g of 8 M NaOH (Sigma-Aldrich, St. Louis, MO, USA). A mass of 16.95 g of sodium silicate solution (Kynita SRL, Barza, Romania) was used to provide an additional supply of reactive silicate species and contribute to the formation of a three-dimensional structure of the (N,K)-A-S-H type. The commercial sodium silicate solution contained 31.10 wt.% SiO_2_ and 13.70 wt.% Na_2_O, corresponding to a SiO_2_/Na_2_O molar ratio of approximately 2.34. Considering the remaining fraction as water, the H_2_O/Na_2_O molar ratio of the sodium silicate solution was estimated to be 13.86.

**Figure 2:**
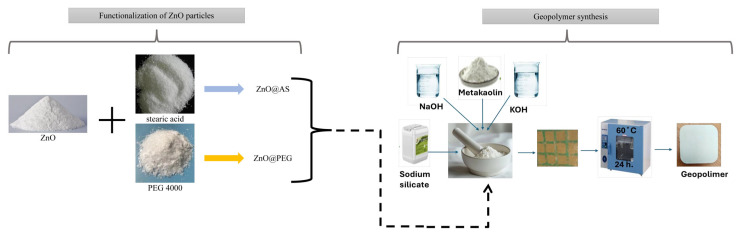
Schematic process of the synthesis of GP.

To obtain the geopolymer samples, 19.5 g of metakaolin was preliminarily homogenized in a dry state with 0.5 g of each type of powder: commercial ZnO, ZnO@AS and ZnO@PEG. The liquid/solid mass ratio was maintained at 1.2 according to the protocol reported in [9]. The calculated Na/Al and K/Al molar ratios were approximately 0.51 and 0.12, respectively, indicating a predominantly sodium-based alkaline activation system with a secondary contribution from potassium species. Such ratios are consistent with the formation of a mixed (N,K)-A-S-H geopolymer network. Therefore, this formulation is particularly suitable for functional geopolymer composites aimed at photocatalytic degradation rather than purely structural performance. The mixing was performed to ensure the most efficient dispersion of the inorganic phase in the final matrix. Subsequently, the alkaline activating solution was gradually added to the solid mixture under continuous mechanical stirring. The sodium silicate solution was added to this paste. The mixing process was maintained until a homogeneous paste was obtained, without visible agglomeration and with a consistency suitable for casting in molds. The resulting paste was cast in cylindrical molds (4 cm diameter) and cured for 24 h at 60 °C in an oven (Biobase Bioland Co., Ltd., Jinan, China), followed by 28 days at room temperature (23 °C) under ambient laboratory conditions, with a measured relative humidity of approximately 10%. The thermal treatment accelerates geopolymer matrix formation, while subsequent curing allows the stabilization of the three-dimensional aluminosilicate network. The initial heat treatment stage had the role of accelerating the dissolution process of the aluminosilicate species and the formation of the geopolymeric matrix, while the subsequent maturation at 23 °C allowed the development and stabilization of the three-dimensional network. During maturation, the aluminosilicate species dissolved from metakaolin react with soluble silicates in the strongly alkaline environment, leading to the formation of an amorphous gel of the (Na,K)-A-S-H type. ZnO particles, regardless of the nature of their surface (unfunctionalized or organically functionalized), are incorporated into the geopolymer matrix as a dispersed phase, contributing to the modification of the structural and functional properties of the final material. A schematic representation of the synthesis procedure is presented in Figure 2.

### 2.2. Methods

Fourier transform infrared (FTIR) spectroscopy in attenuated total reflectance (ATR) mode was applied to identify the principal functional groups of the functionalized ZnO and synthesized geopolymers. Spectra were collected over the 4000–350 cm^−1^ range using a Bruker Tensor 27 spectrometer (Bruker Optik GmbH, Ettlingen, Germany) equipped with a diamond ATR crystal, at a spectral resolution of 4 cm^−1^, with 32 scans averaged per sample.

X-ray diffraction (XRD) analysis was performed using a Rigaku Ultima IV diffractometer (Rigaku Corporation, Tokyo, Japan) operating in Bragg–Brentano (θ–2θ) reflection geometry. The instrument was equipped with a Cu anode X-ray source (Kα_1_ radiation, λ = 1.54178 Å) and a graphite monochromator, operating at 45 kV and 40 mA. The obtained diffraction patterns were interpreted by matching peak positions and intensities with reference data from the PDF 5+ 2024 database to identify the crystalline phases.

The morphology analysis was performed using scanning electron microscopy (SEM) in secondary electron (SE) mode. The samples were fractured, dried, and mounted on aluminum stubs using conductive carbon tape. Elemental composition was analyzed using energy-dispersive X-ray spectroscopy (EDS) attached to the SEM instrument.

SEM and EDS analyses were performed on two different regions of each sample surface to evaluate the spatial homogeneity of the elemental distribution and microstructure.

The textural properties of the geopolymer samples were evaluated using nitrogen adsorption–desorption isotherms at 77 K. The specific surface area was determined by the Brunauer–Emmett–Teller (BET) method, while the pore size distribution and average pore diameter in the mesoporous range were calculated using the Barrett–Joyner–Halenda (BJH) model applied to the desorption branch of the isotherm. The contribution of microporosity and the external surface area were estimated using the t-plot method. The combined application of these approaches provides a comprehensive description of the surface area and pore structure of the investigated geopolymer systems [20].

To highlight the relationships between microstructural and textural parameters, a two-dimensional analysis was conducted, based on scatter plots and linear regression models. Accordingly, the correlations between BET equivalent diameter, pore diameter, BET specific surface area, and total pore volume were investigated through the direct representation of experimental data, avoiding interpolation techniques that might introduce artifacts given the limited number of experimental data points. For each pair of analyzed variables, a linear regression was determined using the Ordinary Least Squares (OLS) method to highlight general variation trends. Simultaneously, for a quantitative assessment of the correlation degree, Pearson and Spearman coefficients were calculated, providing complementary information regarding the existence of linear and monotonic relationships, respectively, between the studied variables.

**Figure 3:**
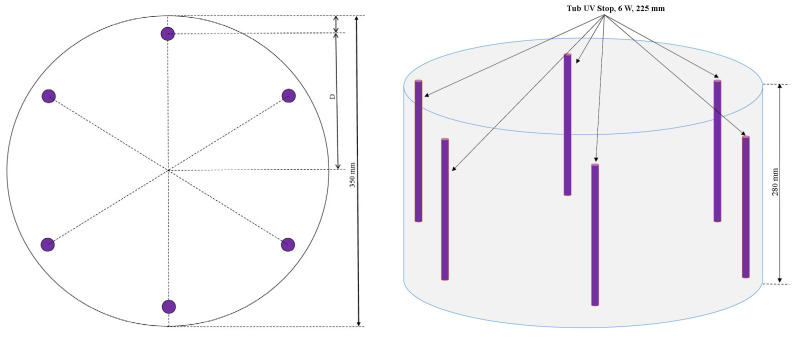
UV reactor.

Methylene blue (MB) (3,7-bis (dimethylamino)-phenothiazin-5-ium chloride) was selected as a model dye to assess photocatalytic activity due to its widespread use in textiles (hair, wool, cotton), paper, and medical applications [4,16,25,26,27]. The photocatalytic efficiency of geopolymers was investigated via MB degradation in aqueous solutions at an initial concentration of 15 ppm. In each experiment, 0.1 g of each MK-based geopolymer was added to 50 mL of MB aqueous solution. The geopolymer samples were ground prior to testing, and no additional particle size classification was applied. However, the textural properties relevant for photocatalytic activity were evaluated by BET analysis, which provides a more accurate representation of the accessible surface area. Prior to UV irradiation, the suspensions were subjected to ultrasonic treatment and maintained in the dark for 30 min, following the protocol previously applied in related geopolymer-based photocatalytic systems reported in the literature [25]. The photocatalytic experiments were subsequently performed under UV, with continuous stirring, for a total irradiation time of 120 min. The UV reactor at lab level consists of an irradiation chamber of cylindrical geometry, used for the controlled exposure of a liquid solution to ultraviolet radiation in the UV-A range, as presented in Figure 3. The enclosure is made of opaque plastic and has the shape of a straight cylinder, with a total height of 28 cm and an inner diameter of 35 cm. Inside the enclosure are mounted six sources of UV-A radiation in the form of actinic fluorescent tubes, each with a nominal electrical power of 6 W and an active length of 225 mm. The tubes are arranged vertically, equidistant angularly, in a circle concentric with the axis of the cylinder, at a constant radial distance from the center. This configuration ensures a symmetrical radial distribution of the ultraviolet radiation field in the interior volume of the enclosure.

The irradiation was from above, and the distance to the suspension surface was 15 cm.

The photocatalytic activity was expressed as MB discoloration efficiency, calculated based on the decrease in absorbance at 664 nm. At periodic intervals of irradiation, a small sample of liquid was removed using a vacuum filter. The removed aliquot was then characterized by UV-Vis spectrophotometry at λ_max_ = 664 nm using a Shimadzu UV-1800 PC UV–visible spectrophotometer (Shimadzu Corporation, Kyoto, Japan). All measurements were carried out in triplicate, and the results are reported as arithmetic mean values. Although small variations are inherent to heterogeneous photocatalytic systems, the results showed good reproducibility, and the differences between samples were significantly larger than the experimental variability. The photocatalytic degradation efficiency (%D) of methylene blue was calculated according to Equation (1):

(1) $$\%D=\frac{A_{0}-A_{t}}{A_{0}}\times 100$$

where $A_{0}$ represents the absorbance measured after establishing adsorption equilibrium prior to UV exposure, and $A_{t}$ corresponds to the absorbance at time t (min).

Assuming first-order kinetics, the degradation process follows the integrated rate equation, obtained from the linear relationship of the natural logarithm of the concentration ratio versus irradiation time [4,25,26,27,28,29].

(2) $$\ln(\frac{C_{o}}{C_{t}})=kt$$

The values of −ln(C/C_0_) were plotted as a function of irradiation time (t) using OriginPro 2021 software, yielding a linear relationship. For a first-order process, the half-life (t_1/2_) is directly related to the rate constant k according to the following expression [12,25,28]:

t_1/2_ = ln2/k (3)

## 3. Results

### 3.1. FTIR Investigation of Surface Functionalization and Matrix Interactions

**Figure 4:**
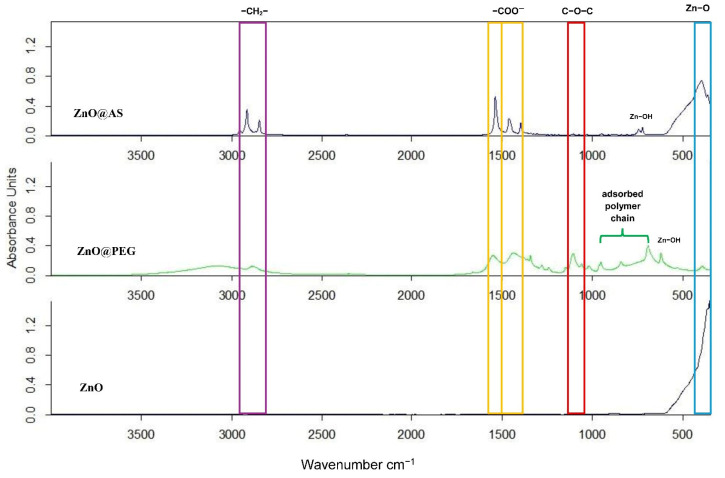
ATR-FTIR spectra of ZnO@AS, ZnO@PEG and ZnO.

The ATR–FTIR spectra of commercial ZnO, stearic acid-functionalized ZnO (ZnO@AS), and PEG-functionalized ZnO (ZnO@PEG) show clear differences in surface chemistry after functionalization, as presented in Figure 4. The spectrum of commercial ZnO is dominated by a strong absorption band in the 430–500 cm^−1^ region, attributed to Zn–O stretching vibrations, confirming the wurtzite structure of ZnO. Weak bands around 3400 cm^−1^ and 1600 cm^−1^ are associated with adsorbed water and surface hydroxyl groups.

For ZnO@AS, additional absorption bands appear, confirming the presence of stearic acid on the ZnO surface. The bands at ~2916 and ~2848 cm^−1^ correspond to asymmetric and symmetric –CH_2_– stretching vibrations, while the bands at ~1540–1560 and ~1400–1420 cm^−1^ are attributed to carboxylate (–COO^−^) groups. The absence or very low intensity of the C=O band near 1700 cm^−1^ suggests that stearic acid is mainly present in its deprotonated form and interacts with Zn^2+^ through ionic or coordinative bonding. The Zn–O band remains visible but with lower intensity, indicating partial coverage of the ZnO surface by the organic layer.

In the ZnO@PEG sample, the spectrum is characterized by a broad band in the 3400–3500 cm^−1^ region corresponding to O–H stretching vibrations and hydrogen-bonded water, bands in the 2870–2930 cm^−1^ region attributed to –CH_2_– stretching vibrations, and a strong band at 1100–1140 cm^−1^ corresponding to C–O–C stretching vibrations, which represents the characteristic spectral signature of PEG. The coexistence of these bands with the Zn–O vibration confirms successful surface functionalization without alteration of the ZnO crystalline structure. The interaction is mainly physical, involving hydrogen bonding and weak surface coordination.

These results confirm that ZnO@AS involves stronger chemical interaction through carboxylate bonding, while ZnO@PEG leads to a more hydrophilic surface modification dominated by physical interactions, which can influence particle dispersion and interfacial compatibility in inorganic matrices.

**Figure 5:**
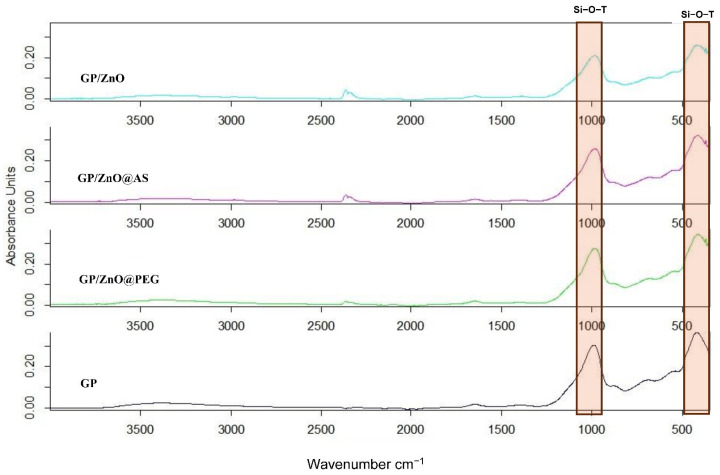
ATR-FTIR spectra of GP/ZnO@AS, GP/ZnO@PEG, GP/ZnO and GP.

The ATR–FTIR spectra of the geopolymer samples exhibit the characteristic features of metakaolin-based geopolymers, together with slight modifications induced by the incorporation of ZnO and functionalized ZnO particles, as presented in Figure 5. All spectra are dominated by the broad band in the 1300–900 cm^−1^ region, corresponding to asymmetric Si–O–T (T = Si, Al) stretching vibrations, characteristic of the amorphous aluminosilicate geopolymer network.

The reference geopolymer (GP) shows the main band centered at approximately 994 cm^−1^. In GP/ZnO, this band shifts slightly toward lower wavenumbers (~982 cm^−1^), indicating a perturbation of the aluminosilicate framework, likely due to interactions between ZnO particles and the geopolymer matrix. In GP/ZnO@AS, the main band appears at ~991–992 cm^−1^, closer to the reference geopolymer, suggesting a more homogeneous dispersion of the functionalized particles and a reduced disturbance of the geopolymer network.

A band around 1645–1650 cm^−1^, assigned to the bending vibration of molecular water, is present in GP and decreases in intensity in ZnO-containing samples, indicating reduced amounts of weakly bound water. In the low-wavenumber region (~420 cm^−1^), bands corresponding to deformation modes of the aluminosilicate lattice are observed in all samples, with possible overlapping contributions from Zn–O vibrations in ZnO-modified geopolymers.

The FTIR results indicate that the incorporation of ZnO does not significantly alter the geopolymer structure but induces local modifications of the aluminosilicate network, with the extent of these modifications depending on the dispersion state and surface functionalization of ZnO particles.

### 3.2. Structural Characterization by XRD

**Figure 6:**
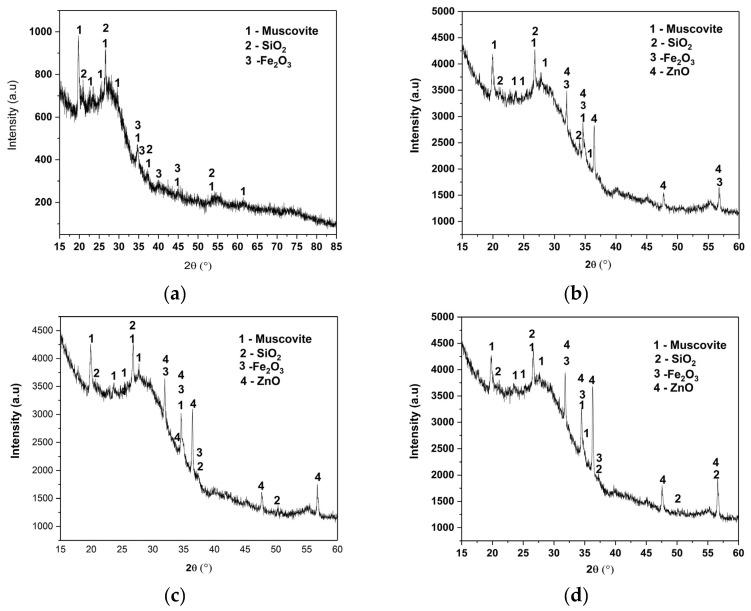
XRD diffractograms of (**a**) GP, (**b**) GP/ZnO, (**c**) GP/ZnO@AS and (**d**) GP/ZnO@PEG.

For the simple geopolymer sample (GP, Figure 6a), qualitative analysis does not reveal well-defined major crystalline phases, indicating a largely amorphous structure characteristic of alkaline-activated metakaolin-based geopolymers (details in Supplementary Materials File S1). 

The diffractogram is dominated by a wide halo, extending approximately between 18° and 35° (2θ), with a diffuse maximum located around 25–28°, associated with the aluminosilicate lattice. Superimposed on the amorphous contribution, discrete crystalline reflections are observed, marked in the figure as belonging to muscovite (KAl_2_(AlSi_3_O_10_) (OH)_2_; according to ICCD PDF-5+ 2025 04-017-7272), silicon dioxide (SiO_2_, quartz; ICCD PDF-5+ 2025 04-007-0522) and iron oxide (Fe_2_O_3_; ICCD PDF-5+ 2025, 01-080-5414). The reflections attributed to quartz (SiO_2_) indicate the presence of a stable, residual crystalline phase from the clay raw material, according to ICCD PDF-5+ 2025 04-007-0522. Quartz does not actively participate in the geopolymerization reaction and remains inert in the alkaline environment, being frequently reported in the literature for metakaolin-based geopolymers. The structural stability of muscovite under alkaline conditions explains its persistence in the final product [1,30]. The relatively low intensity of these reflections compared to the amorphous halo suggests that these crystalline phases have a secondary structural contribution. Reflections attributed to Fe_2_O_3_ (most likely hematite) are also observed, associated with the mineral impurities of the raw material. The low intensities suggest a minor fraction, with no dominant role in the overall structure.

No reflections associated with new crystalline phases (e.g., well-crystallized zeolites) are observed, indicating that geopolymerization predominantly led to the formation of an amorphous structure without significant secondary crystallization. This observation is consistent with the typical mechanism of metakaolin geopolymerization, where the main product is an aluminosilicate matrix.

The diffractogram of ZnO functionalized with stearic acid, ZnO@AS (Figure 6b), also shows a clearly crystalline structure of ZnO (two recordings of ZnO from the ICCD database), along with muscovite reflections, SiO_2_ and Fe_2_O_3_, interpreted as minor secondary phases (details in Supplementary Materials File S1).

In the GP/ZnO, GP/ZnO@AS and GP/ZnO@PEG (Figure 6b–d), on the same amorphous background specific to the geopolymer matrix, there are additional well-defined reflections attributed to the zincite phase (ZnO, hexagonal wurtzite structure), according to the ICCD PDF 5+2025 00-005-0664 used for indexing. These diffraction maxima correspond to characteristic ZnO positions (typically around 31–37° 2θ for planes (100), (002), (101), according to Supplementary Materials File S1), which confirms the presence of crystallized ZnO in geopolymer matrices. In GP/ZnO, zincite reflections are visible but relatively less intense, superimposed on the amorphous halo of the geopolymer. In GP/ZnO@AS, zincite reflections are better defined and better matched (higher ZnO phase figure of merit), suggesting a slightly higher crystallized ZnO content. Functionalization with PEG does not generate detectable new crystalline phases and is compatible with its primary role as a dispersing agent of ZnO particles in the matrix.

The crystal structure of ZnO is preserved after functionalization and integration into the geopolymer.

In all samples where ZnO was detected, the phase was indexed as zincite (ZnO, hexagonal structure) without the appearance of new Zn phases (e.g., Zn(OH)_2_), suggesting that stearic acid is bound at the surface level without altering the oxide crystal lattice. It can be concluded that the amorphous structure of the geopolymer is not strongly affected by ZnO impregnation, and the observed changes are associated with dispersed phases (ZnO, residual muscovite) rather than with a major reorganization of the aluminosilicate lattice. By impregnation with ZnO, either commercially or functionalized with stearic acid, an amorphous–crystalline hybrid composite is obtained, in which the ZnO reflections (zincite) are superimposed on the amorphous halo of the geopolymer.

In conclusion, the XRD analysis confirms the formation of a predominantly amorphous geopolymer matrix, in which residual crystalline phases of quartz and iron oxide from the precursor material are incorporated. The resulting structure is characteristic of metakaolin-based geopolymers and supports the efficiency of the alkaline activation process in generating a continuous aluminosilicate network [30,31,32,33].

### 3.3. SEM-EDS Analysis

**Figure 7:**
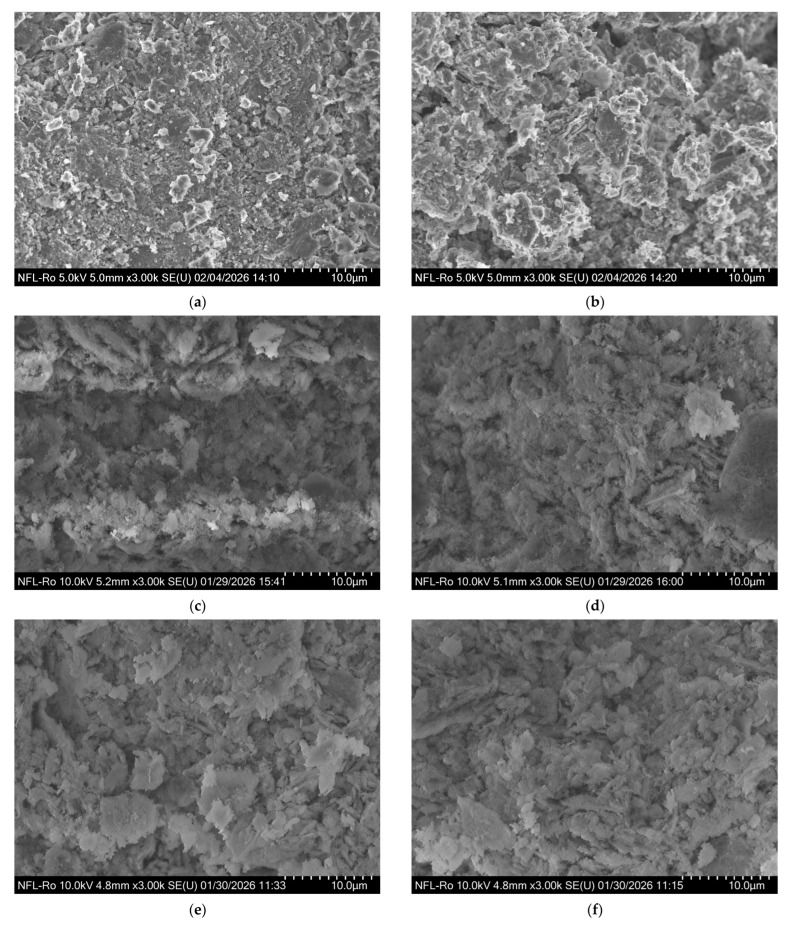

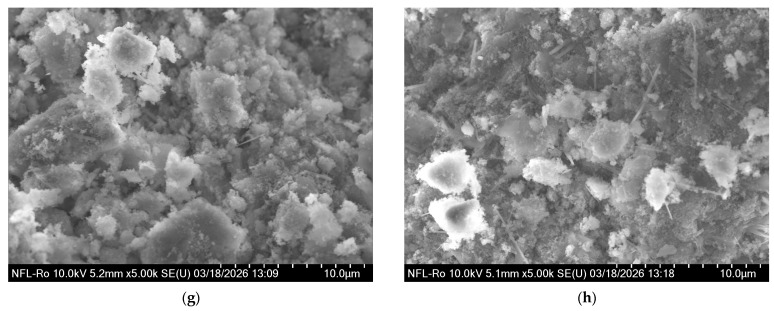
SEM micrographs of (**a**) GP zone 1, ×3000 magnification; (**b**) zone 2, ×3000 magnification; (**c**) GP/ZnO zone 1, ×3000 magnification; (**d**) GP/ZnO zone 2, ×3000 magnification; (**e**) GP/ZnO@AS zone 1, ×3000 magnification; (**f**) GP/ZnO@AS zone 2, ×5000 magnification; (**g**) GP/ZnO@PEG zone 1; and (**h**) GP/ZnO@PEG zone 2, ×5000 magnification.

The comparative analysis of SEM micrographs highlights clear differences between the control geopolymer (GP) and ZnO-modified composites, differences mainly associated with the dispersion state of the semiconductor phase and its interaction with the aluminosilicate matrix, as presented in Figure 7. All measurements were performed on two distinct regions of each sample to describe microstructural homogeneity. Images were acquired in secondary electron (SE) mode at accelerating voltages of 5 kV and 10 kV and a working distance of 4.8–5.2 mm, at magnifications of ×3000 and ×5000. The scale bars correspond to 10 µm.

The simple geopolymer (GP) has a relatively compact and predominantly amorphous microstructure, characterized by a continuous geopolycondensed matrix, with the presence of lamellar particles attributed to partially unreacted metakaolin [1,31]. The phase distribution is homogeneous, and the absence of distinct particles or agglomerations indicates a stable and uniform structure.

In the case of the GP/ZnO composite (commercial ZnO), morphology becomes more heterogeneous. Microagglomerations of ZnO unevenly distributed in the matrix are observed, as well as areas with morphological contrast different from the geopolymeric matrix. Particle agglomeration leads to a more fragmented texture, suggesting an incomplete dispersion of the active phase.

For the GP/ZnO@AS sample, Figure 7e,f indicates an improvement in the uniformity of the particle distribution. The massive agglomerations observed in the case of GP/ZnO are reduced, and the particles are more finely dispersed and more uniformly integrated into the geopolymeric matrix. The GP/ZnO@PEG morphology, as shown in Figure 7g,h, suggests that functionalization with PEG favors the dispersion of ZnO particles in the mass of the geopolymer and limits their coalescence.

### 3.4. EDS Analysis and Elemental Mapping

Energy-dispersive X-ray spectroscopy (EDS) analysis was performed to determine the elemental composition of the geopolymer and ZnO-modified geopolymer samples and to evaluate possible changes in the Si/Al ratio after ZnO incorporation.

**Figure 8:**
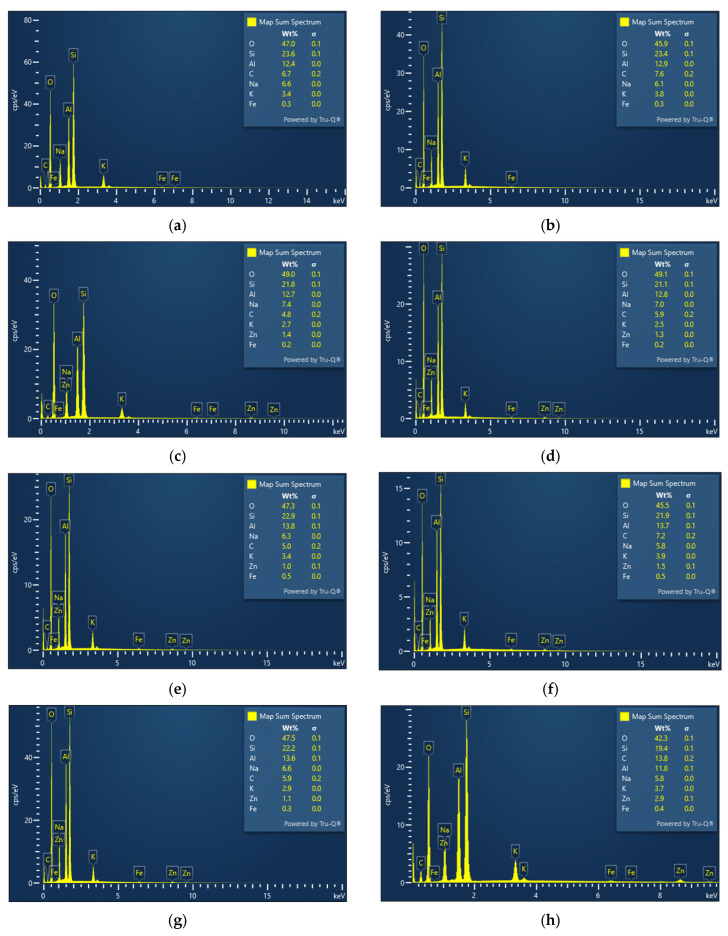
EDS spectra of (**a**) GP zone 1; (**b**) GP zone 2; (**c**) GP/ZnO zone 1; (**d**) GP/ZnO zone 2; (**e**) GP/ZnO@AS zone 1; (**f**) GP/ZnO@AS zone 2; (**g**) GP/ZnO@PEG zone 1; and (**h**) GP/ZnO@PEG zone 2.

For the GP sample, the EDS spectrum (Figure 8a,b) highlights the predominance of the elements O, Si and Al, characteristic of the condensed aluminosilicate matrix, together with Na and K from the alkaline activating solution. The absence of Zn confirms the reference nature of the sample, and the Si/Al ratio is compatible with a stable three-dimensional structure of type (N,K)-A-S-H. In the case of GP/ZnO, the EDS (Figure 8c,d) highlights the presence of Zn (average ~1.35 wt%), confirming the integration of zinc oxide into the matrix. The basic composition of the geopolymer remains stable, without the occurrence of detectable secondary phases. For GP/ZnO@AS, the EDS (Figure 8e,f) confirms the presence of Zn in a similar proportion to that of GP/ZnO, indicating that stearic acid functionalization does not significantly alter the overall Zn content.

The EDS spectrum of GP/ZnO@PEG (Figure 8g,h) confirms the aluminosilicate nature of the geopolymer matrix and the successful incorporation of ZnO. The Zn content (average ~2 wt%) is comparable to that of the other samples, while the Si/Al ratio (~1.63) suggests local dilution effects. Together with the SEM observations, these results support that PEG functionalization mainly affects the dispersion state of ZnO rather than the overall elemental composition of the composite. The Si/Al ratio estimated from the EDS spectra provides an approximate indication of the local composition of the aluminosilicate network, as shown in Table 1.

The EDS-estimated Si/Al ratio decreased progressively after ZnO incorporation, from an average value of 1.86 for the reference geopolymer to 1.69 for GP/ZnO and 1.63 for GP/ZnO@AS, as shown in Table 1.

**Figure 9:**
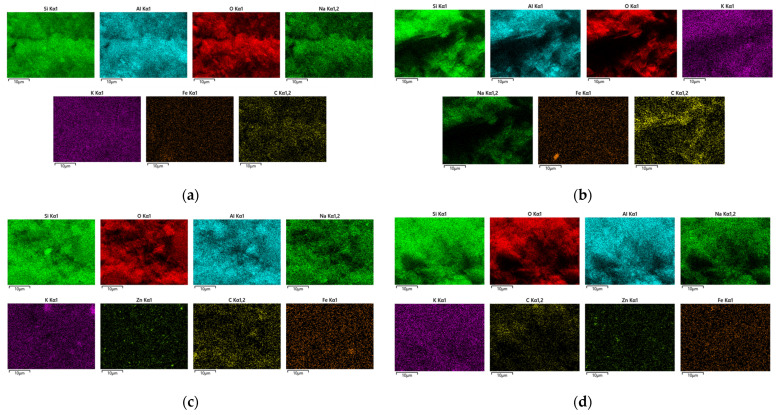

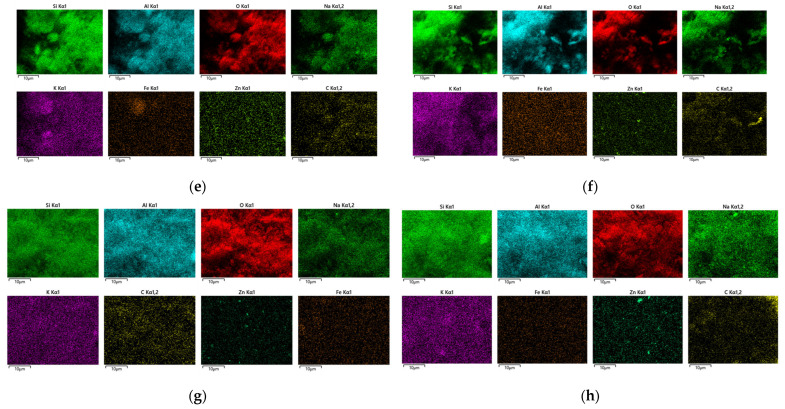
SEM–EDS elemental mapping of geopolymer samples: (**a**,**b**) GP (zones 1 and 2), (**c**,**d**) GP/ZnO (zones 1 and 2), (**e**,**f**) GP/ZnO@AS (zones 1 and 2), and (**g**,**h**) GP/ZnO@PEG (zones 1 and 2).

To further investigate the spatial distribution of Zn within the geopolymer matrix, SEM–EDS elemental mapping was performed for representative samples (Figure 9). The maps corresponding to GP/ZnO, GP/ZnO@AS, and GP/ZnO@PEG systems show that Zn is relatively uniformly distributed across the analyzed regions, without the presence of large agglomerates or phase segregation. At the same time, the distribution of Si, Al, and O remains consistent with the geopolymer matrix, indicating that the incorporation of ZnO does not lead to a disruption of the aluminosilicate framework at the microscale. Minor local variations can be observed, which are typical for heterogeneous porous materials and are associated with local compositional fluctuations and topographical effects.

These results support the interpretation that the variations observed in the Si/Al ratio from point EDS analysis are primarily due to local dilution effects and the surface-sensitive nature of the technique, rather than a structural rearrangement of the geopolymer network.

This compositional evolution is consistent with the structural observations obtained from FTIR analysis, where the main Si–O–T (T = Si, Al) band shows slight shifts, indicating local perturbations of the aluminosilicate framework. In particular, the ZnO-modified geopolymers exhibit a more pronounced shift toward lower wavenumbers, suggesting stronger interactions between ZnO particles and the geopolymer matrix.

### 3.5. BET Analysis

The textural properties of the investigated systems were evaluated by N_2_ adsorption–desorption at 77 K using the BET method for total specific surface area determination, the t-plot method for microporosity assessment, and the BJH model for mesopore distribution analysis, as presented in Table 2 and Table 3.

The reference geopolymer (GP) exhibits the highest specific surface area among all analyzed systems, with a BET value of 36.22 m^2^/g. The single-point surface area (35.41 m^2^/g) is in close agreement with the BET result, indicating a reliable fitting of the adsorption isotherm within the selected relative pressure range. The t-plot analysis reveals a micropore area of 2.71 m^2^/g and an external surface area of 33.51 m^2^/g, demonstrating that the material is predominantly mesoporous, with only a minor but detectable microporous contribution.

The total pore volume determined at P/P_0_ = 0.95 is 0.0939 cm^3^/g (adsorption) and 0.1500 cm^3^/g (desorption), significantly higher than in the ZnO-modified geopolymers. The cumulative BJH pore volume in the 1.7–300 nm range (≈0.279–0.294 cm^3^/g) confirms the presence of a well-developed mesoporous network. These results indicate that the unmodified geopolymer matrix develops a relatively open three-dimensional aluminosilicate framework with interconnected mesopores formed during polycondensation and structural water removal.

In contrast, the incorporation of ZnO leads to a systematic reduction in specific surface area and total pore volume. GP/ZnO exhibits a BET surface area of 13.29 m^2^/g and a total pore volume of 0.0313 cm^3^/g (adsorption), reflecting a moderate development of internal porosity. The average pore diameter determined by BET is approximately 9.43 nm, while BJH analysis indicates the presence of larger mesopores (>30 nm), suggesting a broad pore size distribution. The estimated average BET equivalent diameter (~188 nm) points to partial aggregation and a relatively compact microstructure.

GP/ZnO@AS presents the lowest BET surface area (8.71 m^2^/g) and the smallest total pore volume (~0.0123 cm^3^/g), indicating a significantly reduced porosity. The average pore diameter (≈5.64 nm by BET; ≈7 nm by BJH) suggests a predominance of fine mesopores. The larger average BET equivalent diameter (~276 nm) implies a higher degree of agglomeration, which may limit accessible surface area and partially obstruct the pore network.

GP/ZnO@PEG displays the highest BET surface area among the ZnO-containing samples (17.22 m^2^/g) and a total pore volume of 0.0416 cm^3^/g (adsorption), indicating improved porosity compared to GP/ZnO and GP/ZnO@AS. The average pore diameter (~9.66 nm by BET; >35 nm by BJH) suggests the presence of larger mesopores and a relatively well-preserved porous framework. The smallest estimated BET equivalent diameter (~145 nm) supports the hypothesis of a more efficient dispersion of the ZnO phase within the geopolymeric matrix. The correlation between increased surface area and reduced BET equivalent diameter indicates a more open and accessible microstructure.

The decrease in surface area and pore volume after ZnO incorporation is mainly attributed to pore blocking by ZnO particles and partial densification of the geopolymermatrix.

The magnitude of these effects depends on the dispersion state of ZnO. The GP/ZnO@AS sample exhibits the strongest compaction and the lowest accessible porosity. In contrast, GP/ZnO@PEG retains textural properties closer to the reference GP, suggesting improved interfacial compatibility and a more homogeneous distribution of ZnO that preserves mesopore connectivity. No significant development of additional microporosity is observed in the modified geopolymers, indicating that the overall textural behavior is primarily governed by particle dispersion and interfacial interactions with the geopolymeric matrix.

The results demonstrate that the textural properties of ZnO-modified geopolymers are governed by the dispersion state of the inorganic phase and its interfacial interaction with the aluminosilicate matrix. Uniform dispersion promotes the retention of functional porosity, whereas particle agglomeration leads to densification and reduced accessible surface area.

It is important to emphasize that the BET equivalent diameter derived from BET analysis is not a direct microscopic measurement, but an estimation calculated assuming spherical, non-porous particles using the relation:

$$D_{BET}=\frac{6}{\rho \cdot S_{BET}}$$

where ρ is the density and $S_{BET}$ is the specific surface area. Consequently, the calculated BET equivalent diameter is inversely proportional to the measured surface area.

The GP sample exhibits the smallest estimated BET equivalent diameter (≈69.6 nm), consistent with its highest BET surface area, as presented in Table 4. This indicates a highly developed surface and relatively fine structural organization. The value suggests that the unmodified geopolymer matrix forms smaller structural domains or aggregates with higher accessible surface area.

The incorporation of ZnO results in a substantial modification of the calculated average BET equivalent diameter, as presented in Table 4.

**Figure 10:**
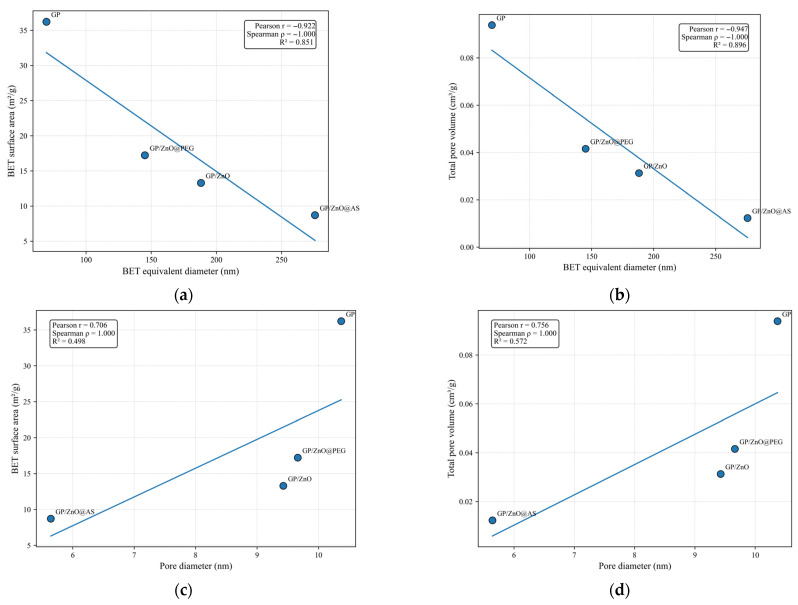
2D representation of (**a**) BET surface area–BET equivalent diameter; (**b**) total pore volume–BET equivalent diameter; (**c**) BET surface area–pore diameter; and (**d**) total pore volume–pore diameter.

The 2D representations from Figure 10 reveal a clear interdependence between these parameters, highlighting the influence of particle size and pore structure on the textural properties of the geopolymer composites. The reference geopolymer (GP) is characterized by the smallest BET equivalent diameter, the largest pore diameter, and consequently the highest BET surface area and total pore volume. This behavior indicates a more open and accessible pore structure, typical for a geopolymeric matrix without filler particles. The incorporation of ZnO particles leads to an increase in BET equivalent diameter and a simultaneous decrease in BET surface area and total pore volume, indicating partial pore filling and structural densification of the geopolymeric matrix. Among the modified samples, GP/ZnO@AS exhibits the largest BET equivalent diameter and the smallest pore diameter, corresponding to the lowest BET surface area and pore volume. This behavior suggests particle agglomeration and partial blocking of pores, leading to a more compact structure.

In contrast, the GP/ZnO@PEG sample shows intermediate BET equivalent diameter, pore diameter, BET surface area, and pore volume values, indicating a more homogeneous dispersion of ZnO particles and a partial preservation of the porous structure. This suggests improved interfacial compatibility between ZnO particles and the geopolymeric matrix in the presence of PEG functionalization.

The 2D correlation analysis demonstrates that the textural properties of the geopolymer composites are controlled by particle size and pore diameter, which are strongly influenced by the dispersion state of ZnO particles and their interaction with the geopolymeric matrix. Increased particle size and reduced pore diameter lead to lower surface area and pore volume, indicating structural densification, while better particle dispersion helps preserve the porous network and accessible surface area.

### 3.6. Photocatalytic Activity

**Figure 11:**
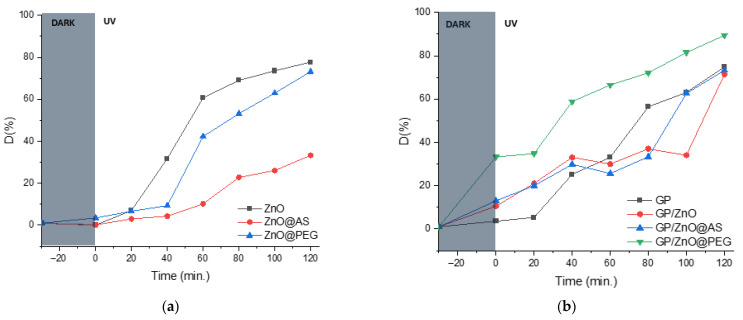
Photocatalytic removal efficiency of MB under UV in the presence of (**a**) ZnO, ZnO@A and ZnO@PEG and (**b**) GP, GP/ZnO@AS and GP/ZnO@PEG.

The evolution of methylene blue (MB) degradation in the presence of the three types of photocatalysts, including the initial ZnO samples, highlights a significant influence of the functionalization of the ZnO surface on the efficiency of the process, as presented in Figure 11. As shown in Figure 9, the initial decrease in MB concentration in the dark (−30 to 0 min) is attributed to adsorption on the material surface. After UV irradiation, a significantly higher removal rate is observed, indicating the dominant contribution of photocatalytic processes.

Commercial ZnO exhibits the highest photocatalytic activity over the entire duration of the experiment, reaching about 60% degradation efficiency after 60 min and about ~78% at 120 min. The marked increase observed in the 40–60 min interval suggests direct accessibility of the active sites and the absence of a passivating organic layer.

ZnO@PEG exhibits intermediate behavior. Although initial degradation is lower (≈10% at 40 min), efficiency increases significantly after 60 min, reaching ~73% at 120 min. This behavior can be correlated with a better dispersion of particles and the reduction in agglomeration phenomena due to the polymer layer, which leads, over time, to a more efficient exposure of the active surface. However, the presence of PEG may initially delay load transfer through a partial surface shielding effect.

In contrast, ZnO@AS exhibits the lowest photocatalytic activity under UV-A, with a degradation of about 10% at 60 min and only ~33% after 120 min. The long hydrocarbon chain of stearic acid gives hydrophobic character to the surface, limiting the interaction between MB (aqueous medium) and the active ZnO sites. In addition, the organic layer can act as a barrier to electron transfer and the generation of oxidative species, which leads to slowed kinetics degradation.

The photocatalytic efficiency follows the order: commercial ZnO > ZnO@PEG >> ZnO@AS.

The results confirm that the nature and properties of the functionalization layer decisively influence the surface accessibility, the load transfer and the oxidative degradation mechanism of the dye, demonstrating that the chemical modification of the ZnO surface can lead to either optimization or inhibition of photocatalytic performance, depending on the hydrophilic/hydrophobic character and the thickness of the organic layer formed.

The photocatalytic removal efficiency of MB under UV-A irradiation highlights a differentiated behavior of geopolymer systems depending on the type of ZnO incorporated and the nature of the functionalization layer.

The control geopolymer (GP) shows a progressive increase in MB conversion, reaching ~75% after 120 min of irradiation. This result indicates that the aluminosilicate matrix contributes to the process through a combined mechanism of adsorption in open porosity and possible limited generation of reactive species on the surface. The more pronounced increase after 60 min suggests an important role of the progressive accumulation of dye in the vicinity of the active areas.

In the case of the GP/ZnO system, a faster degradation is observed (≈33% at 40 min), confirming the photocatalytic activation of ZnO under UV-A. However, at longer irradiation times, the final efficiency (~72% at 120 min) remains slightly lower than the control GP. This behavior can be attributed to imperfect dispersion of ZnO particles in the matrix, local agglomeration or partial shielding of radiation, which reduces the effective accessibility of the semiconductor surface.

The GP/ZnO@AS geopolymer shows a moderate evolution, with a final efficiency close to GP/ZnO (~73% at 120 min). The long hydrocarbon chain of stearic acid gives hydrophobic character to the ZnO surface, limiting the interaction with the aqueous solution of MB and reducing the transfer of charge at the solid–liquid interface. Incorporation into the geopolymer matrix partially mitigates this inhibitory effect through the adsorptive contribution of the substrate but does not lead to a significant improvement in overall performance.

The most effective system is GP/ZnO@PEG, which achieves ~90% degradation after 120 min of UV-A irradiation. The superior performance can be explained by the more uniform dispersion of ZnO particles in the matrix, the reduction in agglomeration and the more hydrophilic character of the PEG layer, which favors contact with the solution and MB access to active sites. Thus, a synergistic effect is outlined between the effect of the porous network of the geopolymer and the generation of reactive species on the ZnO surface, leading to superior degradation kinetics.

The photocatalytic degradation of MB under UV irradiation was evaluated using the pseudo-first-order kinetic model derived from the Langmuir–Hinshelwood mechanism, which is commonly applicable for dilute dye solutions [34,35,36,37].

**Figure 12:**
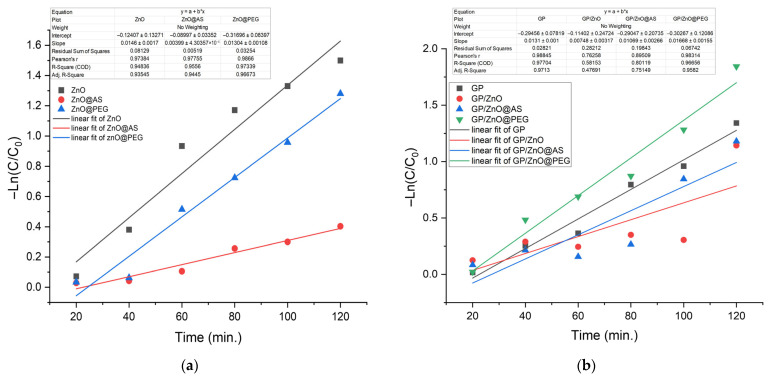
Pseudo-first-order kinetics for photocatalytic degradation and linear fitting of MB in the presence of (**a**) ZnO, ZnO@AS and ZnO@PEG and (**b**) GP, GP/ZnO, GP/ZnO@AS and GP/ZnO@PEG.

The linear fitting of the experimental data of ZnO-based precursors shows correlation coefficients for all investigated systems (R^2^ > 0.94), confirming that the pseudo-first-order model adequately describes the degradation behavior, as presented in Figure 12a. These results reveal a clear kinetic trend:

$$k_{\mathrm{ZnO}}\approx k_{\mathrm{ZnO}@\mathrm{PEG}}\gg k_{\mathrm{ZnO}@\mathrm{AS}}$$

Commercial ZnO exhibits the highest degradation rate, indicating effective generation of reactive oxygen species under UV irradiation and favorable charge separation dynamics. The high linearity confirms that surface reaction kinetics dominate over diffusion limitations.

ZnO@PEG shows a rate constant very close to that of commercial ZnO, suggesting that PEG functionalization does not significantly hinder photocatalytic activity. The hydrophilic nature of PEG likely enhances dispersion and interfacial compatibility in aqueous media, maintaining high accessibility of active sites. The slight reduction in the rate constant compared to bare ZnO may be attributed to partial surface coverage by polymer chains.

In contrast, ZnO@AS presents a noticeably lower apparent rate constant. Although the linear correlation remains high, indicating kinetic consistency, the reduced slope reflects slower degradation. The hydrophobic stearic acid layer likely limits dye adsorption and reduces the effective contact between MB molecules and catalytic active sites.

The analysis of the slopes obtained from the −ln(C/C_0_) representation as a function of time for GP and ZnO-modified GP (Figure 12b) highlights the following order of photocatalytic efficiency:

$$k_{\mathrm{GP}/\mathrm{ZnO}@\mathrm{PEG}}>k_{\mathrm{GP}/\mathrm{ZnO}}>k_{\mathrm{GP}/\mathrm{ZnO}@\mathrm{AS}}>k_{\mathrm{GP}}$$

The GP control sample shows the lowest value of the kinetic constant, indicating a slow degradation of the dye. The observed activity can be attributed mainly to the photocatalytic effects of the aluminosilicate matrix. In the absence of an active semiconductor phase, the generation of reactive species under UV irradiation is limited, which explains the low efficiency.

The introduction of commercial ZnO into the geopolymer matrix leads to a significant increase in the velocity constant. This increase confirms the essential role of ZnO under UV irradiation and in facilitating MB oxidation processes. GP/ZnO exhibits a significantly lower R^2^ value, indicating that its degradation behavior deviates from ideal pseudo-first-order kinetics. This deviation may be attributed to ZnO agglomeration within the geopolymer matrix and reduced accessibility of active sites.

In the case of the GP/ZnO@AS sample, the kinetic constant is lower compared to the commercial ZnO system. Although the pseudo-first-order model is well respected, the speed of reaction is diminished. The hydrophobic character of stearic acid limits the interaction between the water-soluble dye and the catalytic surface, reduces the accessibility of the active centers and can favor the aggregation of particles in the matrix, which leads to lower photocatalytic efficiency.

The GP/ZnO@PEG sample has the highest slope and implicitly the highest apparent speed constant. Functionalization with PEG, having a hydrophilic character, improves the interfacial compatibility between ZnO, the geopolymer matrix and the aqueous environment.

In the case of powders, commercial ZnO exhibits a high kinetic constant, corresponding to a half-life of ~47 min, indicating rapid degradation, as presented in Table 5. Functionalization with PEG maintains comparable activity, suggesting that the hydrophilic layer favors contact with the aqueous environment without significantly blocking the active centers. On the other hand, ZnO@AS shows a sharp decrease in reaction rate, with a much longer half-life, which indicates the inhibition of the photocatalytic process, attributable to the hydrophobic character of stearic acid, which limits MB adsorption and catalytic surface accessibility.

For systems integrated into the geopolymer matrix, kinetic behavior is strongly controlled by the dispersion state and interface compatibility. The GP/ZnO@PEG sample records the highest kinetic constant and the shortest half-life (~41 min), confirming that hydrophilic functionalization with PEG leads to a more uniform dispersion and more efficient exposure of the active centers in the reaction medium. The GP/ZnO@AS sample shows intermediate efficiency, indicating that although stearic acid reduces activity in the case of free powder, in the geopolymer matrix the effect can be partially compensated, probably by a more controlled distribution of particles. Notably, GP/ZnO (commercial ZnO) has the lowest value among the composites, suggesting particle aggregation trapped in the matrix, which reduces the accessibility of the active surface and limits photocatalytic efficiency. The GP control sample shows a relatively high value, which indicates that the geopolymer matrix contributes to the removal of MB through the combination of adsorption and degradation under irradiation, but maximum performance is achieved only when the ZnO phase is introduced in a form that ensures optimal compatibility and dispersion.

The results demonstrate that photocatalytic efficiency, based on UV–Vis decolorization, is determined by a balance between the accessibility of the active surface, the nature of functionalization (hydrophilic vs. hydrophobic), particle aggregation/dispersion and the ZnO–matrix interface interaction. PEG functionalization is the most favorable strategy, leading to the highest values and the lowest half-life for both powders and geopolymer composites, while stearic acid functionalization significantly reduces free-state activity and leads to intermediate behavior after incorporation into the geopolymer.

## 4. Discussion

### 4.1. Correlation of BET–SEM–Photocatalytic Activity

The correlation of the results of BET, SEM and MB photodegradation tests under UV-A irradiation highlights the fact that the performance of geopolymeric systems is determined by the complex interaction between the specific surface, the morphology of the semiconductor phase and the nature of the solid–liquid interface.

The control geopolymer (GP) shows, according to the BET analysis, the highest specific surface area and a low average BET equivalent diameter, indicating a fine structure and a relatively well-developed porosity. SEM confirms a continuous amorphous matrix, with unevenly distributed pores and no dense agglomerations. This structural configuration favors the adsorption of MB in the porous network and the accumulation of the dye in the vicinity of the reactive surfaces. As a result, GP achieves a degradation of approximately 74–75% at 120 min, a performance predominantly attributed to the adsorptive mechanism and progressive oxidation under UV-A, in the absence of dedicated photocatalytic centers.

In the case of GP/ZnO (commercial ZnO), the BET analysis indicates a reduction in the specific surface area and an increase in the BET equivalent diameter compared to GP, suggesting a more compact structure and possible partial obstruction of porosity. SEM images highlight the presence of unevenly dispersed ZnO particles with a tendency to clump. The system shows good initial activity due to the efficient generation of electron–holes in ZnO under UV-A. However, the final efficiency (~71% at 120 min) does not exceed the control GP, indicating that agglomeration reduces the effective active surface area and limits the access of radiation and dye molecules to catalytic sites.

The SEM observations and BET results for the GP/ZnO@AS sample are consistent and indicate a densification of the geopolymeric matrix rather than simple particle agglomeration. SEM images show a relatively uniform distribution of particles within a more compact matrix, while BET measurements reveal a decrease in specific surface area and total pore volume. This behavior suggests that stearic acid functionalization does not primarily lead to large agglomerates but rather promotes matrix densification and partial pore blocking. The hydrophobic organic layer surrounding ZnO particles may reduce the interaction with the alkaline solution and influence the geopolymerization process, leading to a more compact geopolymeric matrix with reduced accessible porosity. Under such conditions, stearic acid can undergo partial saponification, leading to the formation of sodium or potassium stearates. These species may act as surface-active compounds, influencing the rheology of the geopolymer paste and promoting a more compact microstructure. As a result, this process may contribute to matrix densification and partial pore blocking, as reflected in the BET results. However, this interpretation is proposed as a plausible mechanism and would require further investigation for confirmation. Although dispersion is more controlled than in commercial ZnO, the passivating effect of the organic layer leads to a photocatalytic performance like GP and GP/ZnO (~73% at 120 min).

The GP/ZnO@PEG system shows the most favorable correlation between structural parameters and photocatalytic activity. Although the specific area of BET is not necessarily the largest, SEM indicates a more uniform dispersion of particles and a significant reduction in agglomeration. PEG ensures the compatibility of the ZnO phase with the geopolymeric matrix and maintains a more hydrophilic character of the interface, facilitating both MB adsorption and the access of reactive species generated under UV-A. This synergy between the porosity of the geopolymer and the efficient distribution of ZnO leads to the highest degradation (~90% at 120 min).

When correlated with BET and SEM analyses, the kinetic results demonstrate strong consistency. Samples exhibiting higher accessible surface area and improved dispersion (ZnO and ZnO@PEG) show superior photocatalytic performance, whereas systems characterized by aggregation and reduced porosity (ZnO@AS) display diminished activity. Therefore, photocatalytic efficiency is governed not only by the presence of crystalline ZnO but also by surface chemistry, dispersion quality, and accessible active surface area.

The pseudo-first-order kinetic analysis confirms that surface functionalization critically influences photocatalytic behavior. Hydrophilic PEG functionalization preserves catalytic efficiency close to that of unmodified ZnO, while hydrophobic stearic acid functionalization significantly suppresses the degradation rate due to limited surface accessibility and interfacial incompatibility. These findings highlight the importance of interfacial engineering in optimizing ZnO-based photocatalytic systems.

The integrated BET–SEM–photocatalysis analysis demonstrates that performance is not controlled exclusively by the numerical value of the specific area, but by the accessible active area, determined by the degree of dispersion, agglomeration and interfacial properties. The GP/ZnO@PEG system simultaneously optimizes these factors, which explains its superiority in MB degradation under UV-A irradiation.

### 4.2. Correlation of Total Pore Volume–BET–Photocatalytic Efficiency

**Figure 13:**
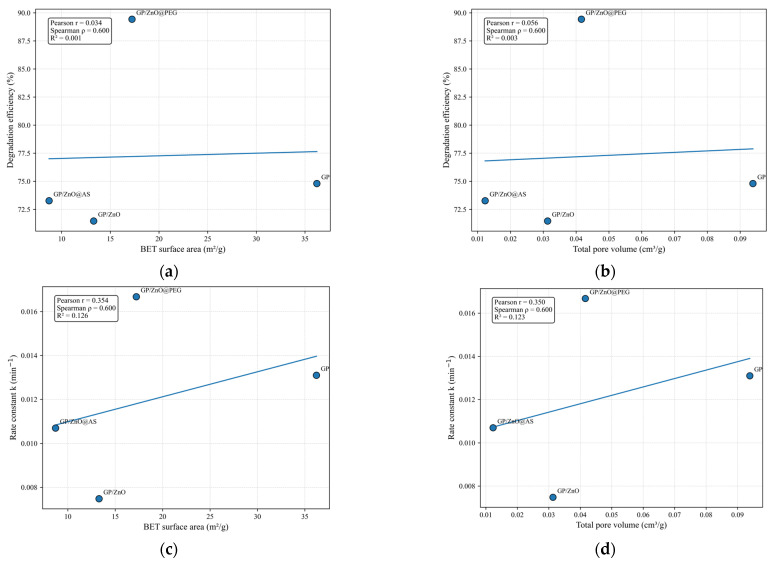
2D representation of (**a**) degradation efficiency–BET surface area; (**b**) degradation efficiency–total pore volume; (**c**) rate constant, k–BET surface area; and (**d**) rate constant, k–total pore volume.

To evaluate the influence of the porous structure on photocatalytic performance, a correlation between the total pore volume, BET and the photocatalytic removal efficiency of MB was analyzed using a 2D representation in Figure 13a.

The results indicate that the total pore volume plays an important role in determining the photocatalytic performance of the geopolymer-based composites, although the relationship is not strictly linear, as shown in Figure 13b. The reference geopolymer (GP) exhibits the highest total pore volume but does not show the highest photocatalytic removal efficiency, indicating that porosity alone is not sufficient to ensure high photocatalytic activity in the absence of photocatalytic particles.

The results show that both total pore volume and BET surface area decrease significantly after ZnO incorporation, indicating partial pore filling, pore blocking, and structural densification of the geopolymeric matrix. The GP/ZnO@AS sample exhibits the lowest BET surface area and the lowest total pore volume, confirming the formation of a more compact structure, most likely due to ZnO particle agglomeration and partial blocking of the porous network.

However, photocatalytic removal efficiency does not follow a simple linear relationship with either BET surface area or total pore volume, as shown in Figure 13c,d. Although the reference geopolymer (GP) shows the highest BET surface area and pore volume, its photocatalytic efficiency is lower than that of the GP/ZnO@PEG sample, indicating that the presence and dispersion of ZnO particles play a more important role than surface area alone.

The GP/ZnO@PEG sample shows intermediate BET surface area and pore volume values but exhibits the highest photocatalytic efficiency. This behavior suggests that an optimal balance between accessible surface area, pore volume, and particle dispersion is achieved in this sample. The preserved porous structure facilitates diffusion of methylene blue molecules, while the improved dispersion of ZnO particles increases the number of accessible photocatalytic active sites.

In contrast, the GP/ZnO and GP/ZnO@AS samples show reduced BET surface area and pore volume due to structural densification and partial pore blocking, which may limit the accessibility of photocatalytic sites and reduce overall photocatalytic performance.

These results demonstrate that photocatalytic efficiency is controlled by the combined effect of surface area, pore volume, and dispersion of ZnO particles within the geopolymer matrix rather than by a single structural parameter, as shown in Figure 11b. The best photocatalytic performance is obtained when a balance between porous structure and particle dispersion is achieved, as observed for the GP/ZnO@PEG sample.

## 5. Conclusions

This study investigated the influence of ZnO surface functionalization on the structural, microstructural, textural, and photocatalytic properties of metakaolin-based geopolymer composites. The results demonstrate that the incorporation of ZnO and functionalized ZnO particles does not significantly alter the predominantly amorphous aluminosilicate network of the geopolymer matrix, as confirmed by XRD and FTIR analyses, but strongly influences the microstructure, porosity, and functional performance of the metakaolin-based geopolymers.

SEM and BET analyses suggest that the behavior of ZnO particles within the geopolymer matrix plays a key role in determining the textural properties. The incorporation of commercial ZnO led to particle agglomeration and partial pore blocking, while stearic acid functionalization promoted matrix densification and reduced accessible porosity. In contrast, PEG-functionalized ZnO is associated with higher BET surface area and more favorable textural properties.

EDS analysis and elemental mapping confirmed the presence of Zn in all modified samples and showed a slight decrease in the local Si/Al ratio after ZnO incorporation, suggesting local compositional effects without altering the overall geopolymer structure.

Photocatalytic tests demonstrated that all ZnO-containing composites exhibited improved methylene blue removal compared to the reference geopolymer. Among the studied materials, the GP/ZnO@PEG showed the highest photocatalytic performance and the largest apparent rate constant, which can be attributed to better interfacial compatibility with the geopolymer matrix and more favorable textural characteristics.

Overall, the results indicate that the performance of ZnO-modified metakaolin-based geopolymers is governed not only by the presence of ZnO but mainly by pore structure, interfacial interactions with the geopolymer matrix, and accessible porosity. Surface functionalization represents an effective strategy for controlling these parameters and optimizing the functional properties of geopolymer-based composite materials. Future work will focus on extending the investigation to visible-light-driven photocatalysis, performing detailed mechanistic studies, including reactive species identification, and assessing the long-term stability, recyclability, and scalability of ZnO-functionalized geopolymer composites.

## Figures and Tables

**Table 1:** Si/Al ratio.

Sample	Si/Al Zone 1	Si/Al Zone 2	Average Si/Al
GP	1.90322	1.81395	1.859
GP/ZnO	1.7165	1.6562	1.686
GP/ZnO@AS	1.6521	1.5985	1.625
GP/ZnO@PEG	1.6232	1.6440	1.633

**Table 2:** BET variation.

Sample	BET (m^2^/g)	Single Point (m^2^/g)	t-Plot Extern (m^2^/g)	BJH ads. (m^2^/g)
GP	36.2189	35.4097	33.5111	31.7441
GP/ZnO	13.2872	12.0841	17.5693	13.9207
GP/ZnO@AS	8.7070	7.6476	12.5120	8.5552
GP/ZnO@PEG	17.2224	15.9127	21.6606	17.7442

**Table 3:** Pore volume variation.

Sample	Vp ads. la P/Po = 0.95 (cm^3^/g)	Vp des. la P/Po = 0.95 (cm^3^/g)	BJH ads. (cm^3^/g)	BJH des. (cm^3^/g)
GP	0.093886	0.150036	0.279365	0.294151
GP/ZnO	0.031313	0.051072	0.115574	0.122103
GP/ZnO@AS	0.012284	0.012958	0.015924	0.014944
GP/ZnO@PEG	0.041598	0.069449	0.171103	0.171277

**Table 4:** Average particle size.

Sample	Adsorption Average Pore Diameter (nm)	Desorption Average Pore Diameter (nm)	BJH Adsorption Average Pore Diameter (nm)	BJH Desorption Average Pore Diameter (nm)	BET Equivalent Diameter (nm)
GP	10.3688	16.5699	35.2021	31.7842	69.6047
GP/ZnO	9.4264	15.3747	33.2095	26.5967	188.1506
GP/ZnO@AS	5.6431	5.9530	7.4455	6.5118	275.6410
GP/ZnO@PEG	9.6614	16.1301	38.5711	28.1498	145.1602

**Table 5:** Kinetic rate and half-life.

Sample	k (min^−1^)	t_1/2_ (min)	Sample	K (min^−1^)	t_1/2_ (min)
ZnO	0.0146 ± 0.0017	47	GP	0.0131 ± 0.001	52
ZnO@AS	0.00399 ± 0.00043	173	GP/ZnO	0.00748 ± 0.00317	92
ZnO@PEG	0.01304 ± 0.0018	53	GP/ZnO@AS	0.01069 ± 0.00266	64
			GP/ZnO@PEG	0.01668 ± 0.00155	41

## Data Availability

Data are contained within the article.

## Supplementary Materials

The following supporting information can be downloaded at: https://www.mdpi.com/article/10.3390/polym18091110/s1, File S1: Analysis Results.
