# Supplementary material for: Influence of Functionalization on the Textural Properties and Photocatalytic Performance of ZnO-Modified Metakaolin Based-Geopolymer
Source: Polymers (Basel). 2026 Apr 30;18(9):1110. doi: 10.3390/polym18091110 (PMC13165240; doi:10.3390/polym18091110)

Analysis Results

General Information

Analysis date	2026/04/24 14:13:14		
Sample name		Measurement date	2025/06/18 10:38:51
File name	Kaolin.raw	Operator	olympus
Comment			

Measurement profile

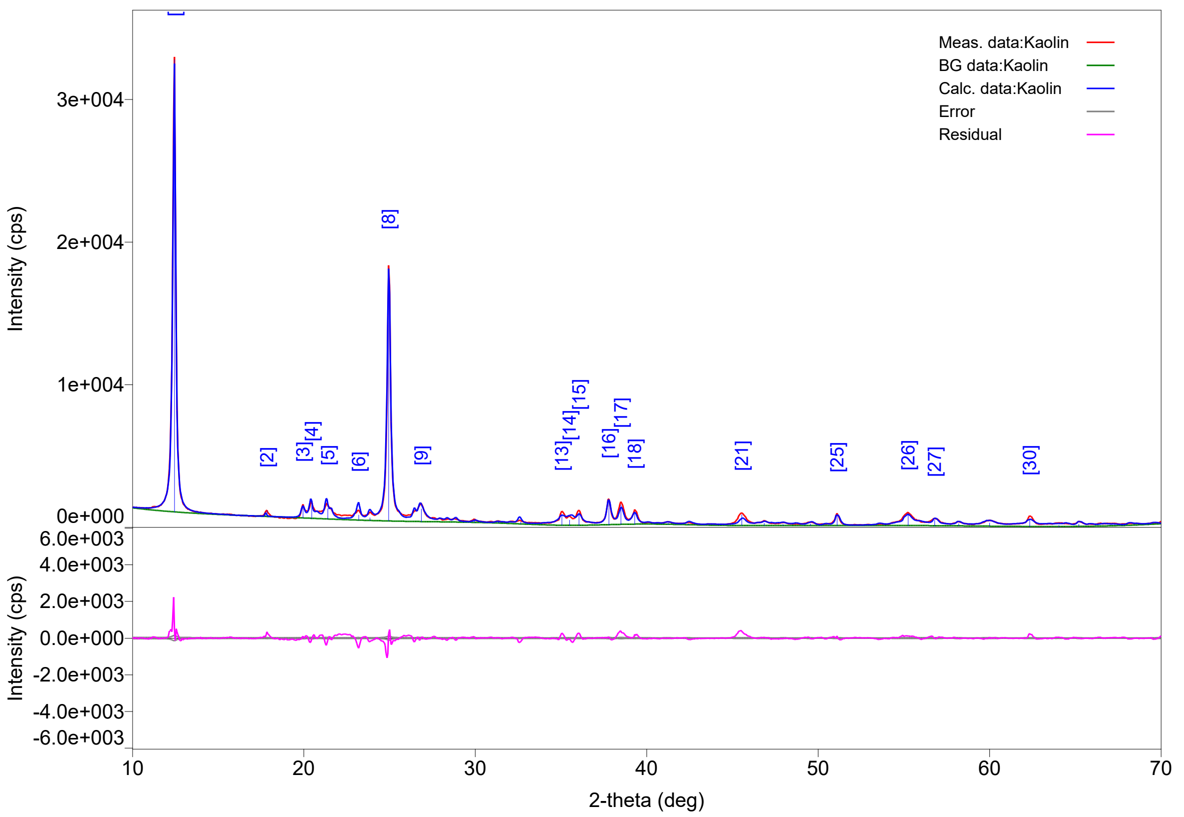

Measurement conditions

X-Ray	45 kV , 40 mA	Scan speed / Duration time	2.0000 deg./min.
Goniometer		Step width	0.0500 deg.
Attachment	-	Scan axis	2theta/theta
Filter		Scan range	10.0000 - 70.0000 deg.
CBO selection slit	-	Incident slit	1.00mm
Diffrected beam mono.		Length limiting slit	-
Detector	D/teX Ultra	Receiving slit #1	Open
Scan mode	CONTINUOUS	Receiving slit #2	Open

Qualitative analysis results

Phase name	Formula	Figure of merit	Phase reg. detail	DB card number
Kaolinite-1A	Al2 Si2 O5 (O H)4	0.479	ICDD (PDF-5+ 2025)	04-013-2830
Muscovite-2M1	K0.932 Al2 (Al0.932 Si3.068	1.043	ICDD (PDF-5+ 2025)	01-086-1384
a-Si O2	Si O2	1.468	ICDD (PDF-5+ 2025)	01-077-1060
Iron Oxide	Fe2 O3	1.579	ICDD (PDF-5+ 2026)	01-085-3769

Phase name	Formula	Space group	Phase reg. detail	DB card number
Kaolinite-1A	Al2 Si2 O5 (O H)4	1 : C1	ICDD (PDF-5+ 2025)	04-013-2830
Muscovite-2M1	K0.932 Al2 (Al0.932 Si3.068	15 : C12/c1,unique-b,cell-1	ICDD (PDF-5+ 2025)	01-086-1384
a-Si O2	Si O2	1 : P1	ICDD (PDF-5+ 2025)	01-077-1060
Iron Oxide	Fe2 O3	63 : Cmcm	ICDD (PDF-5+ 2026)	01-085-3769

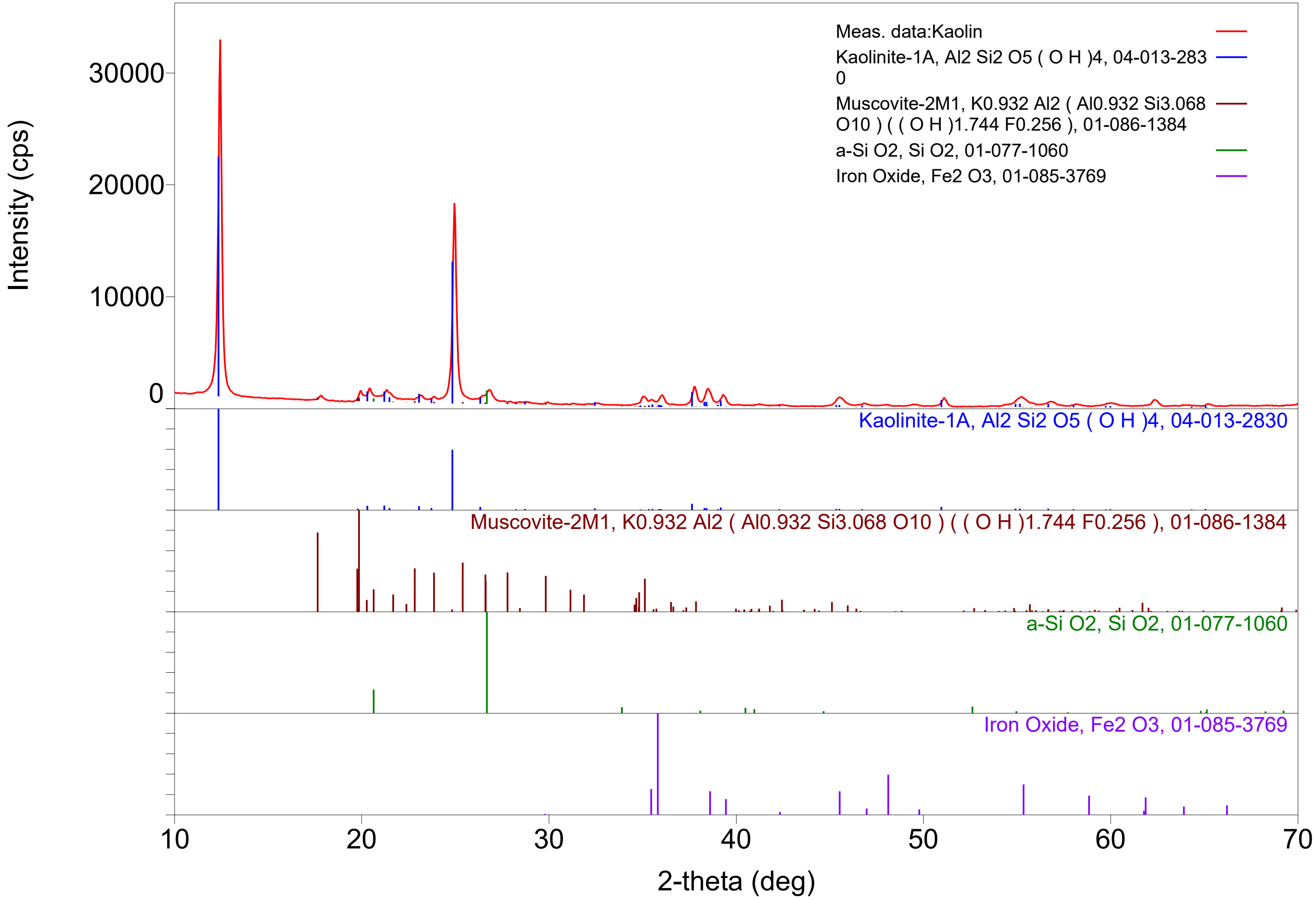
Meas. data:Kaolin
Kaolinite-1A, Al2 Si2 O5 (O H)4, 04-013-283
0
Muscovite-2M1, K0.932 Al2 (Al0.932 Si3.068
O10) ((O H)1.744 F0.256), 01-086-1384
a-Si O2, Si O2, 01-077-1060
Iron Oxide, Fe2 O3, 01-085-3769
Intensity (cps)
30000
20000
10000
0
Kaolinite-1A, Al2 Si2 O5 (O H)4, 04-013-2830
Muscovite-2M1, K0.932 Al2 (Al0.932 Si3.068 O10) ((O H)1.744 F0.256), 01-086-1384
a-Si O2, Si O2, 01-077-1060
Iron Oxide, Fe2 O3, 01-085-3769
10
20
30
40
50
60
70
2-theta (deg)

Peak list

No.	2-theta(deg)	d(ang.)	Height(cps)	Chemical formula	Rel. int. I(a.u.)
1	12.432(3)	7.1141(16)	21807(121)	Al2 Si2 O5 (O H)4	100.00
2	17.81(3)	4.977(9)	288(14)	K0.932 Al2 (Al0.932 Si3.068	2.22
3	19.951(11)	4.447(2)	624(20)	Al2 Si2 O5 (O H)4,K0.932 Al2	3.01
4	20.465(14)	4.336(3)	633(21)	Al2 Si2 O5 (O H)4,K0.932 Al2	5.06
5	21.39(3)	4.150(5)	500(18)	Al2 Si2 O5 (O H)4,K0.932 Al2	6.37
6	23.18(4)	3.834(6)	252(13)	Al2 Si2 O5 (O H)4,K0.932 Al2	1.36
7	23.852(18)	3.727(3)	156(10)	Al2 Si2 O5 (O H)4,K0.932 Al2	0.38
8	24.971(3)	3.5630(4)	13133(94)	Al2 Si2 O5 (O H)4,K0.932 Al2	57.48
9	26.87(3)	3.315(3)	615(20)	Al2 Si2 O5 (O H)4,K0.932 Al2	6.07
10	29.94(3)	2.982(3)	102(8)	K0.932 Al2 (Al0.932 Si3.068	0.31
11	31.36(3)	2.850(2)	72(7)	K0.932 Al2 (Al0.932 Si3.068	0.31
12	32.64(5)	2.741(4)	92(8)	Al2 Si2 O5 (O H)4,K0.932 Al2	1.42
13	35.021(14)	2.5601(10)	571(20)	Al2 Si2 O5 (O H)4,K0.932 Al2	3.38
14	35.446(17)	2.5304(12)	280(14)	Al2 Si2 O5 (O H)4,K0.932 Al2	1.89
15	35.985(14)	2.4937(10)	650(21)	Al2 Si2 O5 (O H)4,K0.932 Al2	4.10
16	37.762(7)	2.3803(4)	1212(28)	Al2 Si2 O5 (O H)4,K0.932 Al2	6.01
17	38.494(10)	2.3367(6)	989(26)	Al2 Si2 O5 (O H)4,K0.932 Al2	7.40
18	39.295(9)	2.2909(5)	618(20)	Al2 Si2 O5 (O H)4,Fe2 O3	3.91
19	41.24(5)	2.187(2)	76(7)	Al2 Si2 O5 (O H)4,K0.932 Al2	0.57
20	42.39(6)	2.130(3)	66(7)	Al2 Si2 O5 (O H)4,K0.932 Al2	0.66
21	45.526(14)	1.9908(6)	548(19)	Al2 Si2 O5 (O H)4,K0.932 Al2	5.99
22	46.826(15)	1.9385(6)	135(9)	Al2 Si2 O5 (O H)4,K0.932 Al2	1.30
23	47.947(18)	1.8958(7)	72(7)	Al2 Si2 O5 (O H)4,K0.932 Al2	0.50
24	49.49(4)	1.8402(15)	96(8)	Al2 Si2 O5 (O H)4,K0.932 Al2	0.75
25	51.069(14)	1.7870(5)	572(20)	Al2 Si2 O5 (O H)4,K0.932 Al2	3.02
26	55.264(17)	1.6608(5)	591(20)	Al2 Si2 O5 (O H)4,K0.932 Al2	10.40
27	56.803(12)	1.6194(3)	306(14)	Al2 Si2 O5 (O H)4,K0.932 Al2	3.03
28	58.18(3)	1.5843(7)	128(9)	Al2 Si2 O5 (O H)4,K0.932 Al2	0.68
29	59.97(4)	1.5413(10)	170(11)	Al2 Si2 O5 (O H)4,K0.932 Al2	1.84
30	62.327(18)	1.4885(4)	469(18)	Al2 Si2 O5 (O H)4,K0.932 Al2	2.67
31	64.00(6)	1.4536(13)	57(6)	Al2 Si2 O5 (O H)4,K0.932 Al2	0.59
32	65.19(2)	1.4300(4)	169(11)	Al2 Si2 O5 (O H)4,K0.932 Al2	0.69

Parameters used for WPPF

Profile parameters

Common parameter	Background	Data	Kaolin
		Function name	B-spline
		param0	1359.0895779367768
		param1	983.49629343654613
		param2	790.2278692459895
		param3	374.69961479412632
		param4	374.76815948621328
		param5	44.712796462758064
		param6	284.86360631721436
		param7	119.99195176857442
		param8	101.83732931459522
		param9	129.11139999765518
		param10	90.52034413796143
		param11	-34.635770148526298
		param12	240.2965078693675
		node0	10
		node1	17.649999999999999
		node2	25.300000000000001
		node3	31.300000000000001
		node4	35.399999999999999
		node5	39.5
		node6	43.600000000000001
		node7	47.700000000000003
		node8	52.899999999999999
		node9	58.25
		node10	70
Common parameter	Peak shift		
		Function name	Shift axial displacement
		param0	0.099999999999732747
		param1	0
		param2	0
Kaolinite-1A	Scale factor	s	15.6(3)

	FWHM	U	0.00(7)
		V	0.08(3)
		W	0.026(3)
	Asym. factor	A0	0.81(6)
		A1	3.4(3)
	Decay rate factor	etaL0/mL0	0.61(3)
		etaL1/mL1	0.6(2)
		etaL2/mL2	0.0000
		etaH0/mH0	1.17(8)
		etaH1/mH1	-0.57(17)
		etaH2/mH2	0.0000
	Preferred orientationMarch-Dollase	h	0
		k	0
		l	1
		March coefficient	0.560(3)
Muscovite-2M1	Scale factor	s	1.02(16)
	FWHM	U	3.05(17)
		V	-0(3)
		W	0.00(3)
	Asym. factor	A0	-1.0(4)
		A1	0(3)
	Decay rate factor	etaL0/mL0	1.5(5)
		etaL1/mL1	0(2)
		etaL2/mL2	0.0000
		etaH0/mH0	1.4(4)
		etaH1/mH1	-0.6(16)
		etaH2/mH2	0.0000
	Preferred orientationMarch-Dollase	h	0
		k	0
		l	0
		March coefficient	1.000000
a-Si O2	Scale factor	s	10.8(12)
	FWHM	U	1(3)
		V	0(8)
		W	0.09(14)
	Asym. factor	A0	0.5(5)
		A1	5(22)

	Decay rate factor	etaL0/mL0	0.0(9)
		etaL1/mL1	-1(19)
		etaL2/mL2	0.0000
		etaH0/mH0	0.8(3)
		etaH1/mH1	1(9)
		etaH2/mH2	0.0000
	Preferred orientationMarch-Dollase	h	0
		k	0
		l	0
		March coefficient	1.000000
Iron Oxide	Scale factor	s	1.9(2)
	FWHM	U	0(88)
		V	-1.0(18)
		W	7(4)
	Asym. factor	A0	2.5(2)
		A1	4.5(15)
	Decay rate factor	etaL0/mL0	1(3)
		etaL1/mL1	-0.4(13)
		etaL2/mL2	0.0000
		etaH0/mH0	0(3)
		etaH1/mH1	-1(7)
		etaH2/mH2	0.0000
	Preferred orientationMarch-Dollase	h	0
		k	0
		l	0
		March coefficient	1.000000

Structure parameters

Data set name	Phase Name	Element	x	y	z	Occupancy	Temperature factor
Kaolin	Kaolinite-1A	Al	0.298600	0.495500	0.475500	1.000	0.500
Kaolin	Kaolinite-1A	Al	0.793700	0.330600	0.474400	1.000	0.500
Kaolin	Kaolinite-1A	Si	-0.003200	0.338300	0.092400	1.000	0.500
Kaolin	Kaolinite-1A	Si	0.510800	0.166800	0.093800	1.000	0.500
Kaolin	Kaolinite-1A	O	0.050300	0.353800	0.316100	1.000	0.500
Kaolin	Kaolinite-1A	O	0.121700	0.662700	0.316600	1.000	0.500
Kaolin	Kaolinite-1A	O	0.000000	0.500000	0.000000	1.000	0.500
Kaolin	Kaolinite-1A	O	0.210300	0.231800	0.024400	1.000	0.500
Kaolin	Kaolinite-1A	O	0.203700	0.763900	0.000300	1.000	0.500
Kaolin	Kaolinite-1A	O	0.050400	0.968700	0.325300	1.000	0.500
Kaolin	Kaolinite-1A	O	-0.041100	0.165700	0.604300	1.000	0.500
Kaolin	Kaolinite-1A	O	0.037300	0.473200	0.604100	1.000	0.500
Kaolin	Kaolinite-1A	O	0.036400	0.856400	0.608000	1.000	0.500
Kaolin	Kaolinite-1A	H	0.142300	1.035300	0.347400	1.000	0.500
Kaolin	Kaolinite-1A	H	0.056000	0.180000	0.701000	1.000	0.500
Kaolin	Kaolinite-1A	H	0.036000	0.486000	0.708000	1.000	0.500
Kaolin	Kaolinite-1A	H	0.033000	0.795000	0.698000	1.000	0.500
Kaolin	Muscovite-2M1	Al	0.251000	0.083800	0.000000	1.000	0.500
Kaolin	Muscovite-2M1	Si	0.451400	0.258200	0.135500	0.767	0.500
Kaolin	Muscovite-2M1	Al	0.451400	0.258200	0.135500	0.233	0.500
Kaolin	Muscovite-2M1	Si	0.034500	0.429500	0.364600	0.767	0.500
Kaolin	Muscovite-2M1	Al	0.034500	0.429500	0.364600	0.233	0.500
Kaolin	Muscovite-2M1	K	0.000000	0.098600	0.250000	0.932	0.500
Kaolin	Muscovite-2M1	O	0.042900	0.061700	0.450100	0.872	0.500
Kaolin	Muscovite-2M1	F	0.042900	0.061700	0.450100	0.128	0.500
Kaolin	Muscovite-2M1	O	0.383600	0.251100	0.053600	1.000	0.500
Kaolin	Muscovite-2M1	O	0.038000	0.444700	0.446300	1.000	0.500
Kaolin	Muscovite-2M1	O	0.412800	0.092500	0.168200	1.000	0.500
Kaolin	Muscovite-2M1	O	0.251600	0.372600	0.168800	1.000	0.500
Kaolin	Muscovite-2M1	O	0.246900	0.308300	0.342600	1.000	0.500

Data set name	Rwp	Rp	Re	S	Chi^2	Maximum shift/e.s.d.
Kaolin	10.97	7.64	2.82	3.8767	15.0284	0.139

Lattice parameters

Angular correction

No correction

Analysis results

Data set name	a(A)	b(A)	c(A)	alpha(deg)	beta(deg)	gamma(deg)
Kaolin	5.164(2)	8.963(4)	7.4095(12)	91.749(17)	104.742(17)	89.82(2)
Kaolin	5.212(6)	8.969(16)	20.185(15)	90.000000	95.89(11)	90.000000
Kaolin	5.29(2)	4.60(3)	5.15(4)	91(2)	89.4(15)	123.6(7)
Kaolin	2.632(13)	9.13(6)	5.994(15)	90.000000	90.000000	90.000000

Phase name	a(A)	b(A)	c(A)	alpha(deg)	beta(deg)	gamma(deg)	V(A^3)
Kaolinite-1A	5.164(2)	8.963(4)	7.4095(12)	91.749(17)	104.742(17)	89.82(2)	331.5(2)
Muscovite-2M1	5.212(6)	8.969(16)	20.185(15)	90.000000	95.89(11)	90.000000	939(2)
a-Si O2	5.29(2)	4.60(3)	5.15(4)	91(2)	89.4(15)	123.6(7)	104.2(12)
Iron Oxide	2.632(13)	9.13(6)	5.994(15)	90.000000	90.000000	90.000000	144.0(12)

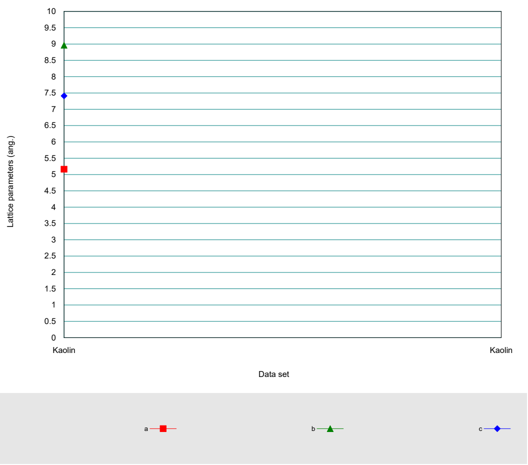

Crystallinity

Data set name	Crystallinity(%)

CrystallinityGraph.emf

Crystallite size and lattice strain

Williamson-Hall method

Data set name	Crystallite size(A)	Strain(%)
Kaolin	350(4)	0.184(18)
Kaolin	757(471)	0.86(9)
Kaolin	274(12)	0.78(3)
Kaolin	26.95(10)	1.69(12)

Phase name	Crystallite size(A)	Distribution RSD	Strain(%)	Distribution type
Kaolinite-1A	350(4)	-	0.184(18)	-
Muscovite-2M1	757(471)	-	0.86(9)	-
a-Si O2	274(12)	-	0.78(3)	-
Iron Oxide	26.95(10)	-	1.69(12)	-

CSSGraph.emf

Quantitative analysis results (RIR)

RIRGraph.emf

Quantitative analysis results (WPPF)

	Phase name	Content(%)
-	Kaolinite-1A	55(2)
-	Muscovite-2M1	9.0(13)
-	a-Si O2	34(3)
-	Iron Oxide	2.6(3)

Quantitative analysis results (standard addition method)

Calibration data

QuantityCalibration.emf

Quantitative analysis results (External Standard method)

Calibration data

QuantityCalibration.emf

Quantitative analysis results (internal standard method)

Calibration Data

QuantityCalibration.emf

Stress

Stress constants

Analytical conditions

Analysis results

StressGraph.emf

Cluster analysis results

Dendrogram

ClusterDendrogram.emf

Measurement profiles

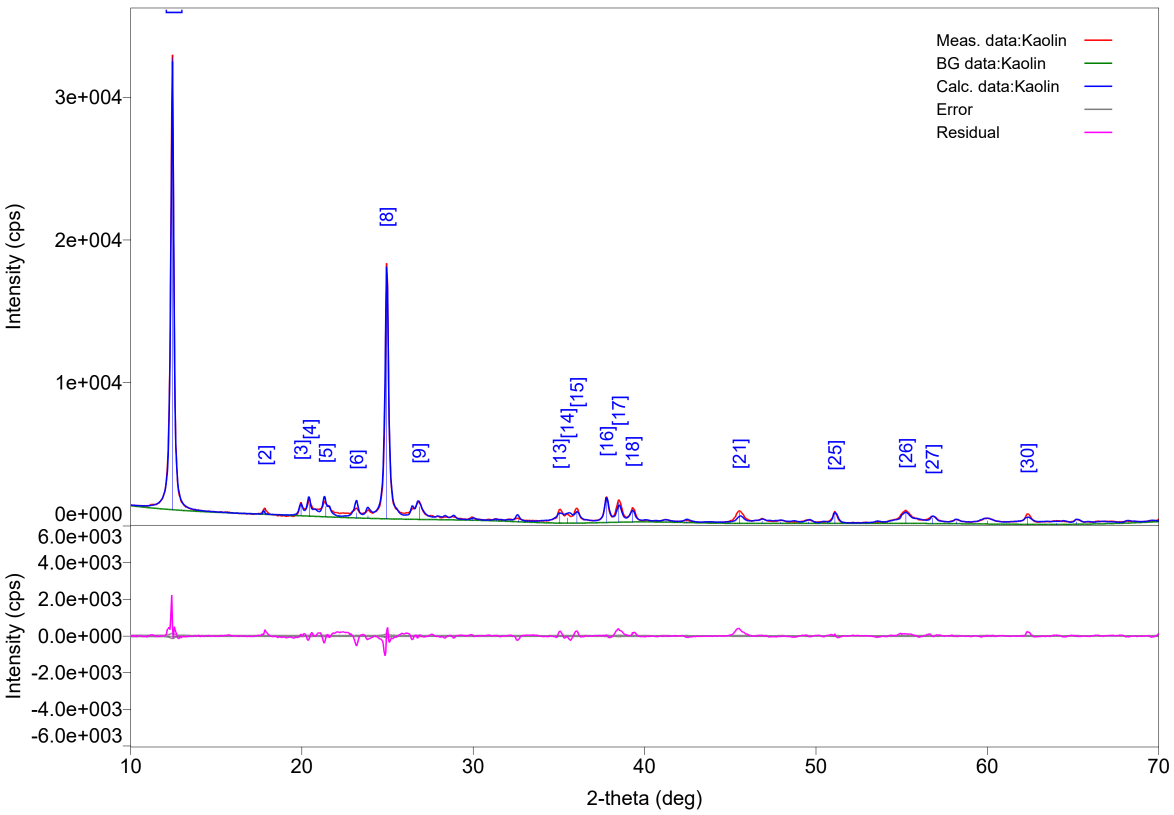

Cluster

Sample well

ClusterSamplePlate.emf

PCA view

ClusterPCA3DGraph.emf

Eigenvalue graph

ClusterPCAGraph.emf

Crystallite size distribution analysis results

Crystallite size distribution

Crystallite size distribution graph

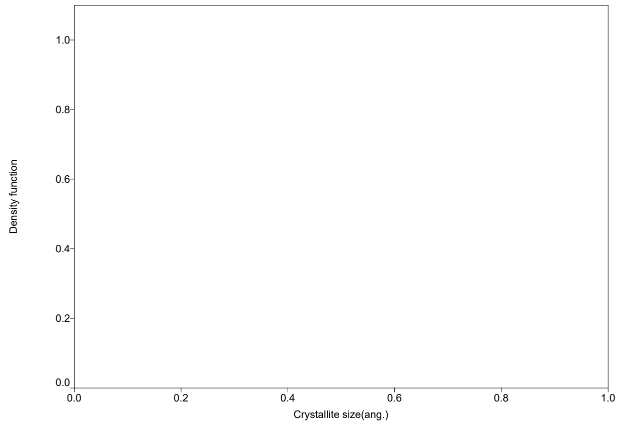

Crystal structure analysis results

Indexing

Phase name	Formula	Figure of merit	Phase reg. detail	DB card number
Kaolinite-1A	Al2 Si2 O5 (O H)4	0.479	ICDD (PDF-5+ 2025)	04-013-2830
Muscovite-2M1	K0.932 Al2 (Al0.932 Si3.068	1.043	ICDD (PDF-5+ 2025)	01-086-1384
a-Si O2	Si O2	1.468	ICDD (PDF-5+ 2025)	01-077-1060
Iron Oxide	Fe2 O3	1.579	ICDD (PDF-5+ 2026)	01-085-3769

Quantitative analysis results

	Phase name	Content(%)
-	Kaolinite-1A	55(2)
-	Muscovite-2M1	9.0(13)
-	a-Si O2	34(3)
-	Iron Oxide	2.6(3)

Lattice information

Phase name	a(A)	b(A)	c(A)	alpha(deg)	beta(deg)	gamma(deg)	V(A^3)
Kaolinite-1A	5.164(2)	8.963(4)	7.4095(12)	91.749(17)	104.742(17)	89.82(2)	331.5(2)
Muscovite-2M1	5.212(6)	8.969(16)	20.185(15)	90.000000	95.89(11)	90.000000	939(2)
a-Si O2	5.29(2)	4.60(3)	5.15(4)	91(2)	89.4(15)	123.6(7)	104.2(12)
Iron Oxide	2.632(13)	9.13(6)	5.994(15)	90.000000	90.000000	90.000000	144.0(12)

Phase name	Space group	Z	Z'	Calc. density(g/cm^3)
Kaolinite-1A	1 : C1	1	0.500	2.586
Muscovite-2M1	15 : C12/c1,unique-b,cell-1	4	0.500	2.791
a-Si O2	1 : P1	3	3.000	2.872
Iron Oxide	63 : Cmcm	4	0.250	7.365

Structure determination

Refinement

Measurement range: 10.0000-70.0000deg   Refinement range: 10.0000-70.0000deg (1.34 A)
Number of refined parameters: 76

Phase name	Atomic coords	# of indep. reflns
Kaolinite-1A	Fractional coords	144
Muscovite-2M1	Fractional coords	199
a-Si O2	-	92
Iron Oxide	-	24

Rwp = 10.97%   S = 3.8767

Crystal structure

CrystalGraph.emf

Analysis Results

General Information

Analysis date	2026/04/24 14:09:45		
Sample name		Measurement date	2025/06/18 10:06:15
File name	MKaolin.raw	Operator	olympus
Comment			

Measurement profile

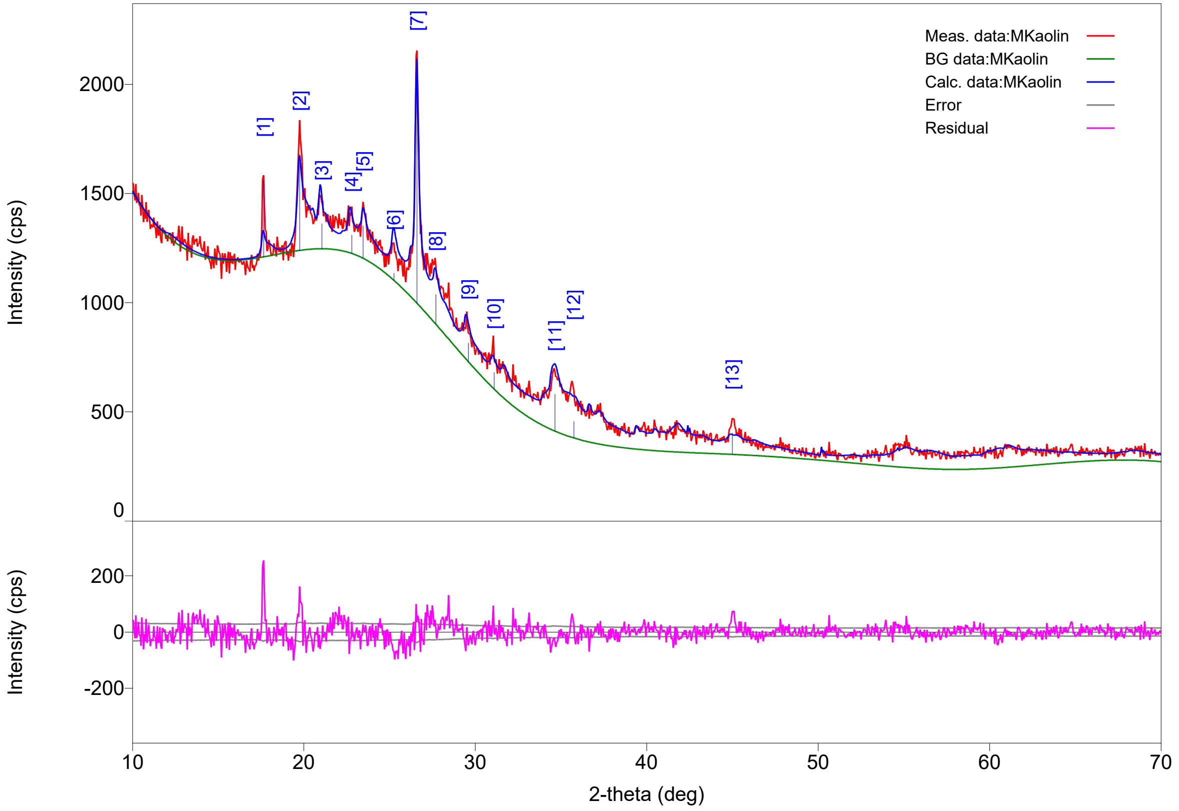

Measurement conditions

X-Ray	45 kV , 40 mA	Scan speed / Duration time	2.0000 deg./min.
Goniometer		Step width	0.0500 deg.
Attachment	-	Scan axis	2theta/theta
Filter		Scan range	10.0000 - 70.0000 deg.
CBO selection slit	-	Incident slit	1.00mm
Diffrected beam mono.		Length limiting slit	-
Detector	D/teX Ultra	Receiving slit #1	Open
Scan mode	CONTINUOUS	Receiving slit #2	Open

Qualitative analysis results

Phase name	Formula	Figure of merit	Phase reg. detail	DB card number
a-Si O2	Si O2	1.253	ICDD (PDF-5+ 2025)	01-077-1060
Muscovite-2M1	K0.932 Al2 (Al0.932 Si3.068	1.384	ICDD (PDF-5+ 2025)	01-086-1384
Kaolinite-1A	Al2 Si2 O5 (O H)4	2.127	ICDD (PDF-5+ 2025)	04-013-2830
Iron Oxide	Fe2 O3	2.844	ICDD (PDF-5+ 2026)	01-085-3769

Phase name	Formula	Space group	Phase reg. detail	DB card number
a-Si O2	Si O2	1 : P1	ICDD (PDF-5+ 2025)	01-077-1060
Muscovite-2M1	K0.932 Al2 (Al0.932 Si3.068	15 : C12/c1,unique-b,cell-1	ICDD (PDF-5+ 2025)	01-086-1384
Kaolinite-1A	Al2 Si2 O5 (O H)4	1 : C1	ICDD (PDF-5+ 2025)	04-013-2830
Iron Oxide	Fe2 O3	63 : Cmcm	ICDD (PDF-5+ 2026)	01-085-3769

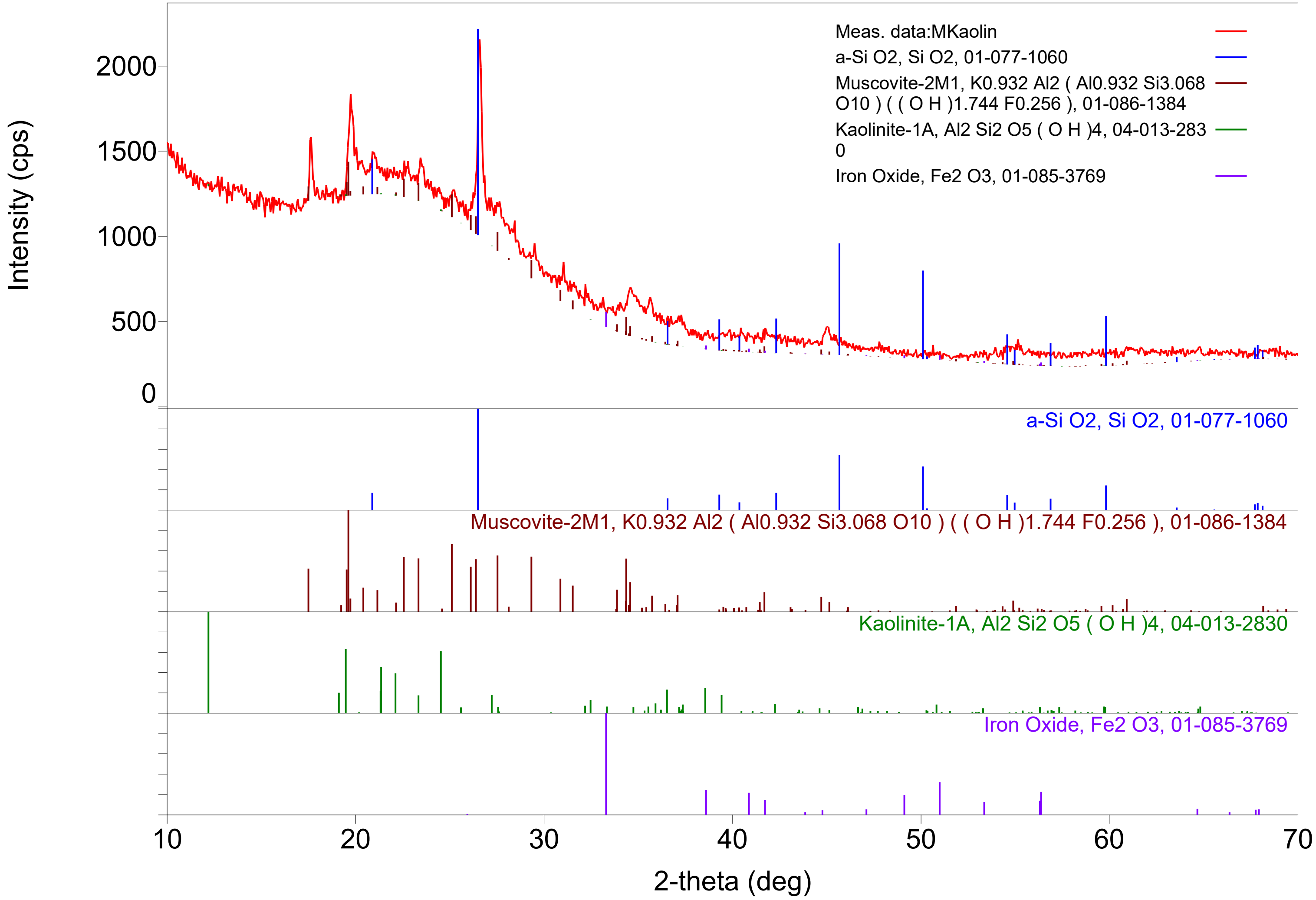

Meas. data:MKaolin
a-Si O2, Si O2, 01-077-1060
Muscovite-2M1, K0.932 Al2 (Al0.932 Si3.068 O10) ((O H)1.744 F0.256), 01-086-1384
Kaolinite-1A, Al2 Si2 O5 (O H)4, 04-013-2830
Iron Oxide, Fe2 O3, 01-085-3769
Intensity (cps)
2000
1500
1000
500
0
a-Si O2, Si O2, 01-077-1060
Muscovite-2M1, K0.932 Al2 (Al0.932 Si3.068 O10) ((O H)1.744 F0.256), 01-086-1384
Kaolinite-1A, Al2 Si2 O5 (O H)4, 04-013-2830
Iron Oxide, Fe2 O3, 01-085-3769
10
20
30
40
50
60
70
2-theta (deg)

Peak list

No.	2-theta(deg)	d(ang.)	Height(cps)	Chemical formula	Rel. int. I(a.u.)
1	17.629(13)	5.027(4)	298(14)	K0.932 Al2 (Al0.932 Si3.068	19.18
2	19.724(14)	4.497(3)	300(14)	K0.932 Al2 (Al0.932 Si3.068	87.80
3	21.0282	4.22124	78.4206	Si O2,K0.932 Al2 (Al0.932	19.79
4	22.7753	3.90123	58.0945	K0.932 Al2 (Al0.932 Si3.068	14.66
5	23.46(3)	3.789(5)	128(9)	K0.932 Al2 (Al0.932 Si3.068	10.50
6	25.232	3.52667	22.1492	K0.932 Al2 (Al0.932 Si3.068	5.59
7	26.572(7)	3.3518(9)	775(23)	Si O2,K0.932 Al2 (Al0.932	100.00
8	27.6888	3.21908	94.5163	K0.932 Al2 (Al0.932 Si3.068	23.85
9	29.5996	3.01547	60.6542	K0.932 Al2 (Al0.932 Si3.068	15.31
10	31.0737	2.87571	54.8972	K0.932 Al2 (Al0.932 Si3.068	13.85
11	34.62(8)	2.589(6)	116(9)	K0.932 Al2 (Al0.932 Si3.068	71.77
12	35.7143	2.51197	54.6028	K0.932 Al2 (Al0.932 Si3.068	13.78
13	44.98(6)	2.014(2)	72(7)	K0.932 Al2 (Al0.932 Si3.068	18.01

Parameters used for WPPF

Profile parameters

Common parameter	Background	Data	MKaolin
		Function name	B-spline
		param0	1511.0772771765648
		param1	915.05593849613911
		param2	1672.5642386284485
		param3	449.50927543056241
		param4	318.35100108864634
		param5	310.0527999964292
		param6	270.47077541314525
		param7	203.74531686633739
		param8	300.21459308151594
		param9	271.85941364474144
		node0	10
		node1	23.300000000000001
		node2	32
		node3	38.549999999999997
		node4	45.100000000000001
		node5	51.649999999999999
		node6	58.25
		node7	70
Common parameter	Peak shift		
		Function name	Shift axial displacement
		param0	0.06934076839461413
		param1	0
		param2	0
a-Si O2	Scale factor	s	4.7(3)
	FWHM	U	0.7(2)
		V	-0.63(17)
		W	0.15(3)
	Asym. factor	A0	0.3(9)
		A1	1(4)
	Decay rate factor	etaL0/mL0	0.0(4)

		etaL1/mL1	-1(6)
		etaL2/mL2	0.0000
		etaH0/mH0	0.9(3)
		etaH1/mH1	1(2)
		etaH2/mH2	0.0000
	Preferred orientationMarch-Dollase	h	0
		k	0
		l	0
		March coefficient	1.000000
Muscovite-2M1	Scale factor	s	2.33(14)
	FWHM	U	5.0(5)
		V	-0.3(2)
		W	0.00(14)
	Asym. factor	A0	-1.5(2)
		A1	0.0(13)
	Decay rate factor	etaL0/mL0	1.3(5)
		etaL1/mL1	0.6(2)
		etaL2/mL2	0.0000
		etaH0/mH0	1.54(16)
		etaH1/mH1	0.27(8)
		etaH2/mH2	0.0000
	Preferred orientationMarch-Dollase	h	0
		k	0
		l	0
		March coefficient	1.000000
Kaolinite-1A	Scale factor	s	0.6(4)
	FWHM	U	2(139)
		V	3(27)
		W	3(6)
	Asym. factor	A0	-1.5(19)
		A1	0(13)
	Decay rate factor	etaL0/mL0	0(8)
		etaL1/mL1	1(15)
		etaL2/mL2	0.0000
		etaH0/mH0	1(3)
		etaH1/mH1	0(3)
		etaH2/mH2	0.0000

	Preferred orientationMarch-Dollase	h	0
		k	0
		l	0
		March coefficient	1.000000
Iron Oxide	Scale factor	s	2.0(16)
	FWHM	U	5(39)
		V	1(2)
		W	1(37)
	Asym. factor	A0	0.5(13)
		A1	0(17)
	Decay rate factor	etaL0/mL0	0.9(13)
		etaL1/mL1	-1(13)
		etaL2/mL2	0.0000
		etaH0/mH0	0(5)
		etaH1/mH1	-1(44)
		etaH2/mH2	0.0000
	Preferred orientationMarch-Dollase	h	0
		k	0
		l	0
		March coefficient	1.000000

Structure parameters

Data set name	Phase Name	Element	x	y	z	Occupancy	Temperature factor
MKaolin	Muscovite-2M1	Al	0.251000	0.083800	0.000000	1.000	0.500
MKaolin	Muscovite-2M1	Si	0.451400	0.258200	0.135500	0.767	0.500
MKaolin	Muscovite-2M1	Al	0.451400	0.258200	0.135500	0.233	0.500
MKaolin	Muscovite-2M1	Si	0.034500	0.429500	0.364600	0.767	0.500
MKaolin	Muscovite-2M1	Al	0.034500	0.429500	0.364600	0.233	0.500
MKaolin	Muscovite-2M1	K	0.000000	0.098600	0.250000	0.932	0.500
MKaolin	Muscovite-2M1	O	0.042900	0.061700	0.450100	0.872	0.500
MKaolin	Muscovite-2M1	F	0.042900	0.061700	0.450100	0.128	0.500
MKaolin	Muscovite-2M1	O	0.383600	0.251100	0.053600	1.000	0.500
MKaolin	Muscovite-2M1	O	0.038000	0.444700	0.446300	1.000	0.500
MKaolin	Muscovite-2M1	O	0.412800	0.092500	0.168200	1.000	0.500
MKaolin	Muscovite-2M1	O	0.251600	0.372600	0.168800	1.000	0.500
MKaolin	Muscovite-2M1	O	0.246900	0.308300	0.342600	1.000	0.500
MKaolin	Kaolinite-1A	Al	0.298600	0.495500	0.475500	1.000	0.500
MKaolin	Kaolinite-1A	Al	0.793700	0.330600	0.474400	1.000	0.500
MKaolin	Kaolinite-1A	Si	-0.003200	0.338300	0.092400	1.000	0.500
MKaolin	Kaolinite-1A	Si	0.510800	0.166800	0.093800	1.000	0.500
MKaolin	Kaolinite-1A	O	0.050300	0.353800	0.316100	1.000	0.500
MKaolin	Kaolinite-1A	O	0.121700	0.662700	0.316600	1.000	0.500
MKaolin	Kaolinite-1A	O	0.000000	0.500000	0.000000	1.000	0.500
MKaolin	Kaolinite-1A	O	0.210300	0.231800	0.024400	1.000	0.500
MKaolin	Kaolinite-1A	O	0.203700	0.763900	0.000300	1.000	0.500
MKaolin	Kaolinite-1A	O	0.050400	0.968700	0.325300	1.000	0.500
MKaolin	Kaolinite-1A	O	-0.041100	0.165700	0.604300	1.000	0.500
MKaolin	Kaolinite-1A	O	0.037300	0.473200	0.604100	1.000	0.500
MKaolin	Kaolinite-1A	O	0.036400	0.856400	0.608000	1.000	0.500
MKaolin	Kaolinite-1A	H	0.142300	1.035300	0.347400	1.000	0.500
MKaolin	Kaolinite-1A	H	0.056000	0.180000	0.701000	1.000	0.500
MKaolin	Kaolinite-1A	H	0.036000	0.486000	0.708000	1.000	0.500
MKaolin	Kaolinite-1A	H	0.033000	0.795000	0.698000	1.000	0.500

Data set name	Rwp	Rp	Re	S	Chi^2	Maximum shift/e.s.d.
MKaolin	4.07	3.1	3.03	1.339	1.7929	2.073

Lattice parameters

Angular correction

No correction

Analysis results

Data set name	a(A)	b(A)	c(A)	alpha(deg)	beta(deg)	gamma(deg)
MKaolin	4.911(4)	4.920(9)	5.436(6)	89.05(15)	90.37(8)	119.67(6)
MKaolin	5.245(6)	9.219(10)	20.36(2)	90.000000	96.01(6)	90.000000
MKaolin	5.19(14)	9.3(3)	7.8(2)	95.1(18)	111.7(15)	84.5(16)
MKaolin	2.42(3)	8.65(15)	6.86(10)	90.000000	90.000000	90.000000

Phase name	a(A)	b(A)	c(A)	alpha(deg)	beta(deg)	gamma(deg)	V(A^3)
a-Si O2	4.911(4)	4.920(9)	5.436(6)	89.05(15)	90.37(8)	119.67(6)	114.1(3)
Muscovite-2M1	5.245(6)	9.219(10)	20.36(2)	90.000000	96.01(6)	90.000000	978.9(19)
Kaolinite-1A	5.19(14)	9.3(3)	7.8(2)	95.1(18)	111.7(15)	84.5(16)	349(18)
Iron Oxide	2.42(3)	8.65(15)	6.86(10)	90.000000	90.000000	90.000000	144(4)

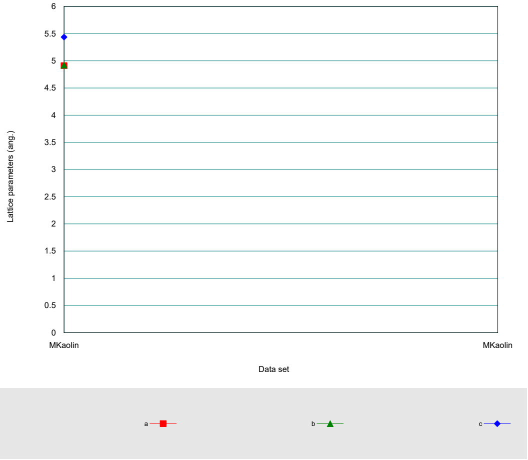

Crystallinity

Data set name	Crystallinity(%)

CrystallinityGraph.emf

Crystallite size and lattice strain

Williamson-Hall method

Data set name	Crystallite size(A)	Strain(%)
MKaolin	233(4)	0.25(5)
MKaolin	100000.000000	2.78(7)
MKaolin	32.86(14)	2.03(7)
MKaolin	72.39(4)	0.601(7)

Phase name	Crystallite size(A)	Distribution RSD	Strain(%)	Distribution type
a-Si O2	233(4)	-	0.25(5)	-
Muscovite-2M1	100000.000000	-	2.78(7)	-
Kaolinite-1A	32.86(14)	-	2.03(7)	-
Iron Oxide	72.39(4)	-	0.601(7)	-

CSSGraph.emf

Quantitative analysis results (RIR)

RIRGraph.emf

Quantitative analysis results (WPPF)

	Phase name	Content(%)
-	a-Si O2	39(3)
-	Muscovite-2M1	50(4)
-	Kaolinite-1A	6(4)
-	Iron Oxide	5(4)

Quantitative analysis results (standard addition method)

Calibration data

QuantityCalibration.emf

Quantitative analysis results (External Standard method)

Calibration data

QuantityCalibration.emf

Quantitative analysis results (internal standard method)

Calibration Data

QuantityCalibration.emf

Stress

Stress constants

Analytical conditions

Analysis results

StressGraph.emf

Cluster analysis results

Dendrogram

ClusterDendrogram.emf

Measurement profiles

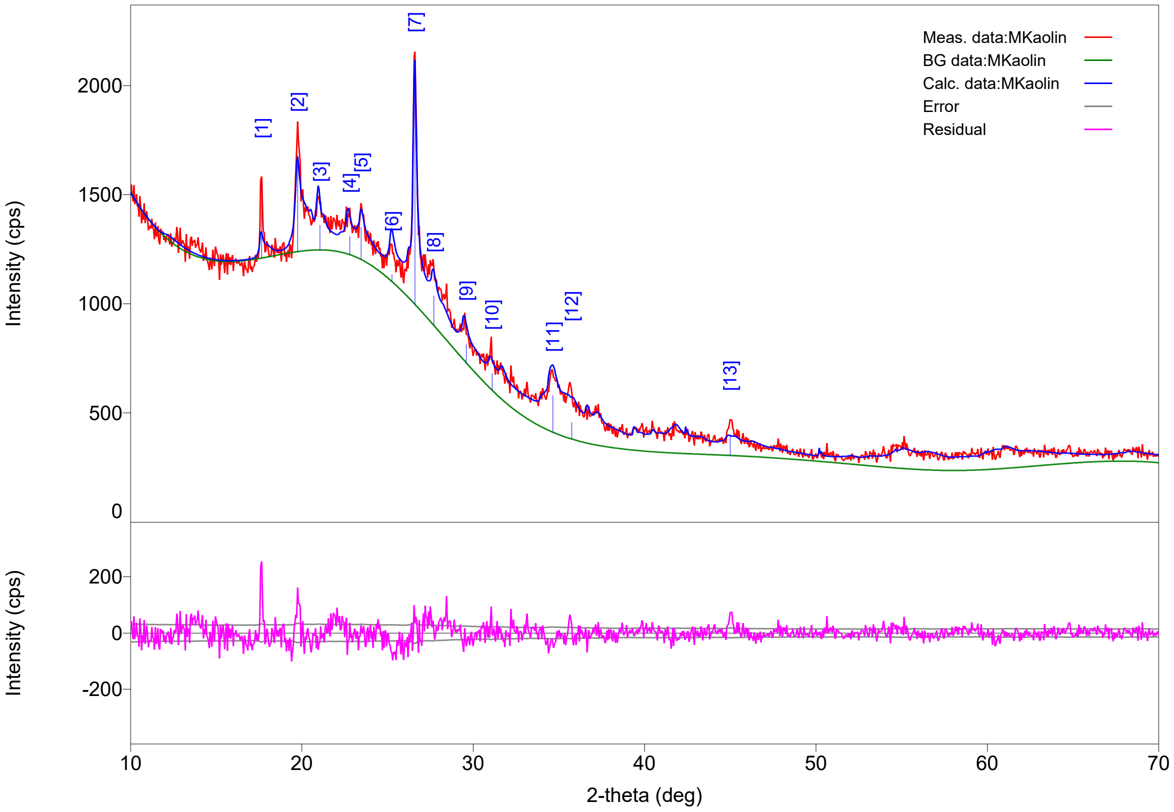

Cluster

Sample well

ClusterSamplePlate.emf

PCA view

ClusterPCA3DGraph.emf

Eigenvalue graph

ClusterPCAGraph.emf

Crystallite size distribution analysis results

Crystallite size distribution

Crystallite size distribution graph

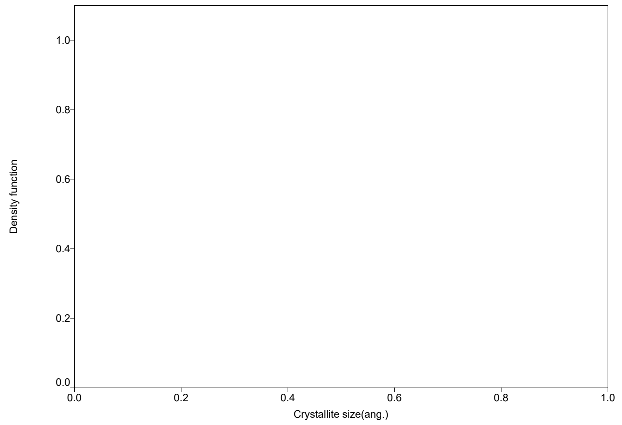

Crystal structure analysis results

Indexing

Phase name	Formula	Figure of merit	Phase reg. detail	DB card number
a-Si O2	Si O2	1.253	ICDD (PDF-5+ 2025)	01-077-1060
Muscovite-2M1	K0.932 Al2 (Al0.932 Si3.068	1.384	ICDD (PDF-5+ 2025)	01-086-1384
Kaolinite-1A	Al2 Si2 O5 (O H)4	2.127	ICDD (PDF-5+ 2025)	04-013-2830
Iron Oxide	Fe2 O3	2.844	ICDD (PDF-5+ 2026)	01-085-3769

Quantitative analysis results

	Phase name	Content(%)
-	a-Si O2	39(3)
-	Muscovite-2M1	50(4)
-	Kaolinite-1A	6(4)
-	Iron Oxide	5(4)

Lattice information

Phase name	a(A)	b(A)	c(A)	alpha(deg)	beta(deg)	gamma(deg)	V(A^3)
a-Si O2	4.911(4)	4.920(9)	5.436(6)	89.05(15)	90.37(8)	119.67(6)	114.1(3)
Muscovite-2M1	5.245(6)	9.219(10)	20.36(2)	90.000000	96.01(6)	90.000000	978.9(19)
Kaolinite-1A	5.19(14)	9.3(3)	7.8(2)	95.1(18)	111.7(15)	84.5(16)	349(18)
Iron Oxide	2.42(3)	8.65(15)	6.86(10)	90.000000	90.000000	90.000000	144(4)

Phase name	Space group	Z	Z'	Calc. density(g/cm^3)
a-Si O2	1 : P1	3	3.000	2.623
Muscovite-2M1	15 : C12/c1,unique-b,cell-1	4	0.500	2.677
Kaolinite-1A	1 : C1	1	0.500	2.454
Iron Oxide	63 : Cmcm	4	0.250	7.385

Structure determination

Refinement

Measurement range: 10.0000-70.0000deg Refinement range: 10.0000-70.0000deg (1.34 A)
Number of refined parameters: 72

Phase name	Atomic coords	# of indep. reflns
a-Si O2	-	106
Muscovite-2M1	Fractional coords	201
Kaolinite-1A	Fractional coords	145
Iron Oxide	-	24

Rwp = 4.07% S = 1.3390

<u>Crystal structure</u>

CrystalGraph.emf

Qualitative Analysis Results

General information

Analysis date	2026/03/04 11:15:12		
Sample name		Measurement date	2025/10/06 11:24:08
File name	GP.raw	Operator	olympus
Comment			

Qualitative analysis results

Phase name	Formula	Figure of merit	Phase reg. detail	DB card number
a-Si O2, quartz-alpha	Si O2	1.261	ICDD (PDF-5+ 2025)	04-007-0522
Muscovite	H2 K Al3 Si3 O12	1.822	ICDD (PDF-5+ 2025)	00-002-0058
Iron Oxide	Fe2 O3	1.523	ICDD (PDF-5+ 2025)	01-085-3769

Phase data pattern

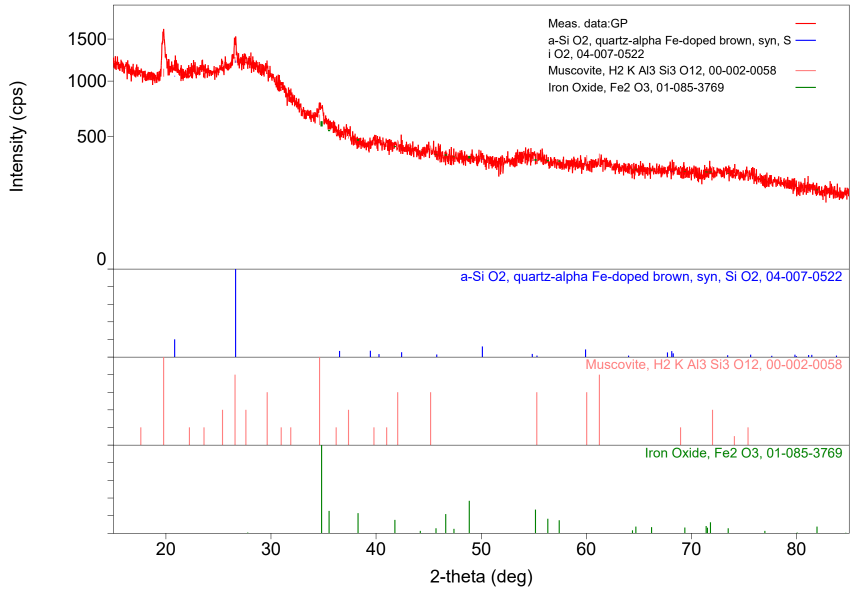

Qualitative Analysis Results

General information

Analysis date	2026/03/04 11:19:19		
Sample name		Measurement date	2025/10/07 09:22:52
File name	GP_ZnO_1.raw	Operator	olympus
Comment			

Qualitative analysis results

Phase name	Formula	Figure of merit	Phase reg. detail	DB card number
Zincite, syn	Zn O	0.515	ICDD (PDF-5+ 2025)	00-005-0664
muscovite	K Al3 Si3 O11	1.222	ICDD (PDF-5+ 2025)	04-017-7272
Iron Oxide	Fe2 O3	2.920	ICDD (PDF-5+ 2025)	01-085-3769
Quartz	Si O2	1.489	ICDD (PDF-5+ 2025)	01-091-6075

Phase data pattern

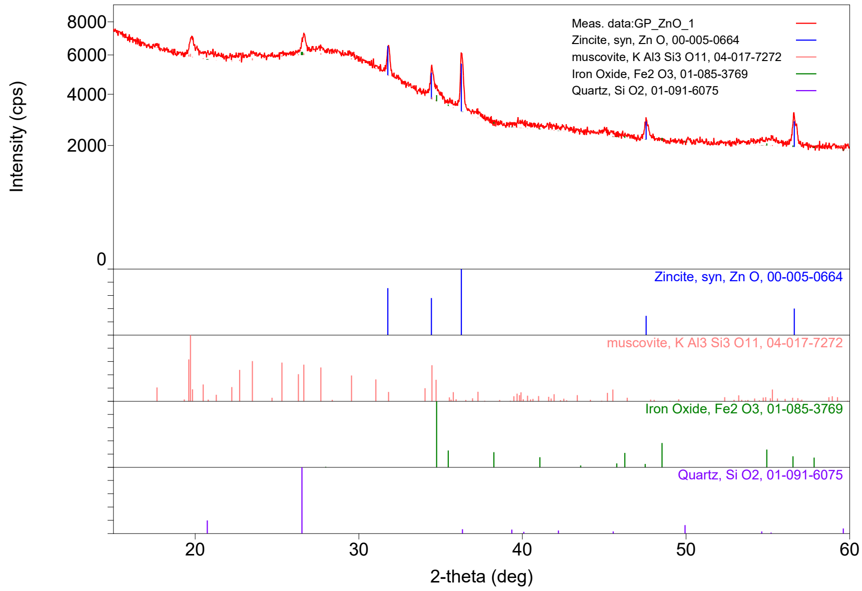

Qualitative Analysis Results

General information

Analysis date	2026/03/04 11:22:38		
Sample name		Measurement date	2025/10/07 09:49:42
File name	GP_ZnO_2.raw	Operator	olympus
Comment			

Qualitative analysis results

Phase name	Formula	Figure of merit	Phase reg. detail	DB card number
muscovite	K Al3 Si3 O11	1.494	ICDD (PDF-5+ 2025)	04-017-7272
Zincite, syn	Zn O	0.944	ICDD (PDF-5+ 2025)	00-005-0664
a-Fe2 O3, hematite,	Fe2 O3	1.365	ICDD (PDF-5+ 2025)	04-018-0098
a-Si O2, quartz-alpha	Si O2	1.602	ICDD (PDF-5+ 2025)	04-007-0522

Phase data pattern

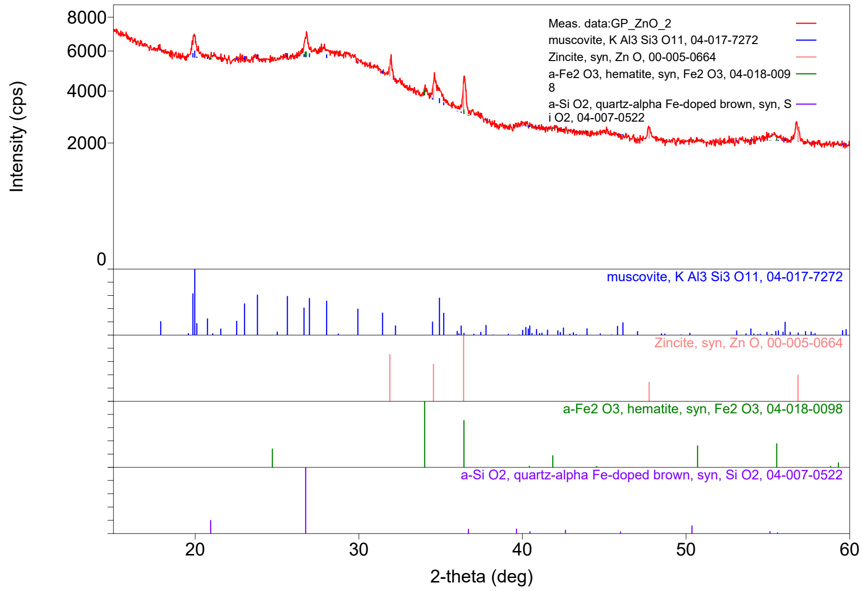

Qualitative Analysis Results

General information

Analysis date	2026/03/04 11:24:09		
Sample name		Measurement date	2025/10/07 10:15:22
File name	GP_ZnO_3.raw	Operator	olympus
Comment			

Qualitative analysis results

Phase name	Formula	Figure of merit	Phase reg. detail	DB card number
Zincite, syn	Zn O	0.922	ICDD (PDF-5+ 2025)	00-005-0664
muscovite	K Al3 Si3 O11	1.483	ICDD (PDF-5+ 2025)	04-017-7272
a-Fe2 O3, hematite,	Fe2 O3	1.900	ICDD (PDF-5+ 2025)	01-080-5414
a-Si O2, quartz-alpha	Si O2	1.452	ICDD (PDF-5+ 2025)	04-007-0522

Phase data pattern

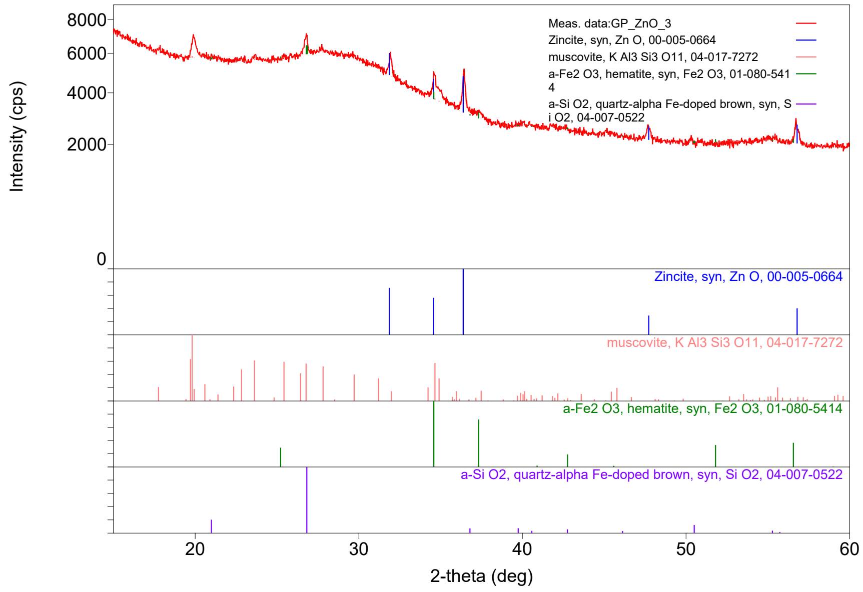

Supplement: Supplementary file 1 [file polymers-18-01110-s001.zip › polymers-4277981-supplementary.pdf]
